# Epoxy Resins for Negative Tone Photoresists

**DOI:** 10.3390/polym11091457

**Published:** 2019-09-06

**Authors:** Vitor Vlnieska, Andrey Mikhaylov, Margarita Zakharova, Eva Blasco, Danays Kunka

**Affiliations:** 1 Karlsruhe Institute of Technology (KIT), Institute of Microstructure Technology, Hermann-von-Helmholtz-Platz 1, 76344 Eggenstein-Leopoldshafen, Germany; 2 Federal University of Paraná, Chemistry Department, Rua Coronel Francisco Heráclito dos Santos, 100, Jardim das Américas, Curitiba 81531-980, PR, Brazil; 3 Karlsruhe Institute of Technology (KIT), Institute for Chemical Technology and Polymer Chemistry (ITCP), Engesserstr. 18, 76131 Karlsruhe, Germany

**Keywords:** lithography, deep X-ray lithography (DXRL), deep ultraviolet lithography (DUVL), RMN, ESI-µ-TOF-MS, DSC, SEM-EDX

## Abstract

One of the types of negative tone photoresists is composed of at least a catalyst, a solvent, and epoxy resin. This is the primary raw material for lithography technology. To ensure high-quality pattern transfer in the lithography process, it is crucial to control the properties of the photoresist. In this work, a set of resins based on Bisphenol-A were synthesized. The obtained resins have been characterized regarding the chain size and its derivative products. As a second step, an epoxidation reaction was performed and the epoxy groups were quantified. The profile of the resins, obtained by mass spectroscopy (ESI-µ-TOF-MS), showed that it is possible to tune the chain sizes of the polymers and their derivate by controlling the parameters of the polymerization reaction. Three profiles of resins were achieved in this study. Nuclear magnetic resonance (NMR) indicates an epoxidation in the range of 96%, when comparing the phenolic peak intensity before and after the reaction. Differential Scan Calorimetry (DSC) measurements confirmed the different oligomer profiles of resins, showing different glass transition temperatures.

## 1. Introduction

Lithographic techniques such as deep X-ray lithography (DXRL), X-ray lithography (XRL), deep ultraviolet lithography (DUVL) and ultraviolet lithography (UVL) rely on photo reactive materials named photoresists. Although these materials were developed nearly 40 years ago [1], there is still a lack of data concerning their mechanical and chemical properties, as well as the overall performance of the material during lithography fabrication related to the chemical composition of photoresists. A class of negative tone epoxy-based photoresists, also known as SU8 formulation, is widely applied in DXRL and DUVL [2,3,4,5,6,7,8]. In this work, polymerization synthesis was studied and optimized by applying factorial planning. The polymerization degree of the bisphenol-A is fundamental to defining the mechanical properties of the resins that make up the photoresist. By varying the polymerization parameters (such as the temperature, molar ratio between reagents, and reaction time), it is possible to control and tune the chain sizes, as well as their distribution, and make a prediction regarding the amount of secondary and perhaps tertiary products generated from them [9,10]. Depending on the monomer and reagents (starting materials), this reaction can be difficult to control, resulting in a wide range of possible products.

The forward reaction, alkylation of the hydroxyl groups, also known as an epoxidation step, is mainly responsible for providing the curing effect, a crosslinking reaction (together with a photocatalyst) during the exposure step in the lithography techniques. The epoxidation procedure is widely applied for several chemical products [9,10], and the control and characterization of this reaction are also necessary, since the epoxy content, together with the amount of catalyst, define the photosensitivity of the photoresist. All these factors together, the polymerization, epoxidation, formulation with the photocatalyst, and a solvent, generate a polymer/resin with unique characteristics, generating a fingerprint that varies for each batch.

**Figure 1:**
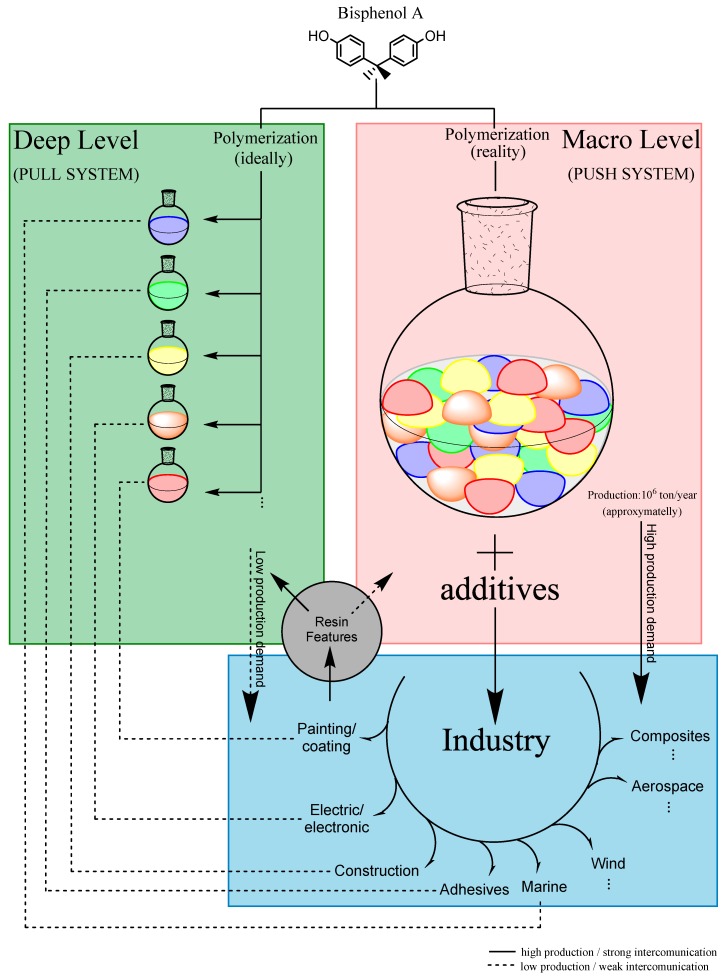
Micro and macro levels of tuning and synthesis of the resins.

Regarding the composition and formulation of the photoresists, two levels of tuning properties can be considered, represented in Figure 1:

1: Deep levels—optimize the synthesis parameters of the resins, leading to specific chemical and mechanical properties (green part of Figure 1);
2: Macro-level—tune the features of the resins previously synthesized by introducing other chemical compounds to the formulation (red part of Figure 1).

For this case, when the whole synthesis process of the photoresists is not well-defined, the complexity of the raw material can present an infinite number of possible characteristics. For instance, it will be interesting to divide this process into two main parts, as mentioned by “Deep” and “Macro” levels.

The Deep level is crucial to standardizing the synthesis of the polymer (or at least an attempt) as close as possible to the desired properties. For epoxy negative photoresists based on Bisphenol-A, it is essential to elucidate at least two reactions: standardization of the polymerization step and standardization of the epoxidation step. These are the main challenges to defining the final properties of the resin that will make up the photoresist. The Deep level is commonly performed at a laboratory scale.

The Macro level represents the formulation of the photoresists, mixing the resin previously synthesized with at least two more necessary compounds: a photocatalyst and a solvent. Other chemical compounds in this step are also commonly added to tune the overall properties of the mixture, and these components are widely known in the industry field as resinous modifiers (influencing properties like flexibility, toughness, peel strength, adhesion, etc.), fillers, colorants and dyes, and other additives (e.g., rheological additives, flame retardants) [11]. The Macro level is commonly performed in scaled-up production (industry). 

## 2. Materials and Methods 

### 2.1. Materials

The chemicals and consumables were purchased from Sigma-Aldrich (Darmstadt, Germany). Bisphenol-A (≥99%), p-formaldehyde (≥94.0%), epichlorohydrin (≥99%), and Boron trifluorideethearate -BF3 content: 46%–51% (BF_3_Et_2_O) were the main chemicals used for the synthesis step. Triarylsulfonium salts (TAS) (50 wt % in propylene carbonate) and polar solvents such as ketones, isopropanol (IPA) (anhydrous, 99.5%), and dimethyl sulfoxide (max. 0.025% H_2_O), as well as the home-made resins, were the main chemicals used to perform the formulation of the photoresists. All the chemicals were used as received.

### 2.2. Polymerization of Bisphenol-A

In a 50 mL one-necked flask, Bisphenol-A and a solution of NaOH/formaldehyde were added. The system was connected to a reflux condenser. Different parameters, such as the concentration of the NaOH/formaldehyde solution, molar ratio Bisphenol-A: formaldehyde, temperature, time, and pressure, as well as the presence of solvent, were studied (see Table S1 in the supplementary material for detailed information regarding the values of the parameters). After the reaction was complete, the residual water was removed by distillation. The products were then characterized by the reported methods. 

### 2.3. Alkylation of the Phenolic Groups

In a 100 mL one-necked flask, oligomers were added in the molar ratio 1:25 (oligomers:THF) with the solvent. After the solution was complete, a molar ratio 1:0.06 (oligomers:BF_3_Et_2_O) was added to the mixture. After one hour under constant heating at 45 °C, a molar ratio of 1:20 (oligomers:epichlorohydrin) was slowly and carefully added to the reaction, over circa 4 h, where, after 3 h of adding epichlorohydrin, the reaction system had a temperature that had increased to 70 °C. The reaction system was cooled to room temperature and an alkali solution was added, with the molar ratio 1:2.1 (oligomers:alkali), and the reaction system was heated to 60 °C for 3 h. After cooling down to room temperature, the reaction mixture was neutralized with an acid solution until the pH was near to 7. The molar ratio 1:175 (oligomers:H_2_O) of water was used to wash the solution (four times 1:175). The organic layer was separated, and the solvent was evaporated. The products obtained were characterized by the reported methods.

### 2.4. Electrospray Ionization-Time of Flight-Mass Spectroscopy (ESI-µTOF-MS)

The samples were prepared in a concentration range of 10^−6^ mol·mL^−1^ and solubilized in acetone (2% NaCl (*m*/*v*)). The spectra were recorded on a micrOTOF-QII spectrometer (Bruker, Karlsruhe, Germany). The acquisition was set to a positive mode, 5.5 × 10^3^ V, no pressure at the nebulizer, dry gas flow at 3.0 mL·mL^−1^, a dry temperature of 90 °C, a transfer system with radio frequency (RF) 1 and RF 2 at 200 VPP, Hexapole at 100 VPP, an ion energy of 3.0 eV, a collision energy at 12.0, collision RF of 250 VPP, a transfer time of 70 µs, and pre-storage of 5.0 µs. The mass range was initially recorded from 1 × 10^2^ to 1 × 10^4^ m·z^−1^, after not observing any peaks in the high-mass region, and the spectra were recorded in a range from 1 × 10^2^ to 2.5 × 10^3^ m·z^−1^.

### 2.5. Nuclear Magnetic Resonance Spectroscopy (NMR)

All the samples were prepared in acetone-d_6_, with a range of 40 mg of mass. The experiments, such as protons (^1^H), carbon (^13^C-exp. comp. pulse decoupling), and Heteronuclear Single Quantum Coherence (HSQC), were performed using the standard parameters, predetermined by Bruker′s software (Karlsruhe, Germany,). The proton experiments were acquired with 124 scans and carbon experiments with 1024 scans using Bruker 500 MHz equipment (Bruker, Karlsruhe, Germany).

### 2.6. Differential Scan Calorimetry (DSC)

#### Oligomer′s Measurements 

The samples were weighted in a range of 40 mg. The analyses were performed in a DSC 30 (Mettler-Toledo Instruments, Giessen, Germany). The samples were evaluated following the heating procedure:

1: Heated until 180 °C, with a heating flow of 10 °C·min^−1^;
2: 3 min isotherm;
3: Cooled until −50 °C, with a cooling flow of 10 °C·min^−1^;
4: Heated until 180 °C, with a heating flow of 10 °C·min^−1^;
5: Cooled until 25 °C, with a cooling flow of 10 °C·min^−1^.

#### Epoxidized Oligomer′s Measurements 

The samples were weighted in a range of 40 mg. The measurements were performed in a DSC 30 (Mettler-Toledo Instruments, Giessen, Germany). The samples were evaluated following the heating procedure:

1: Cooled until −150 °C, with a cooling flow of 10 °C·min^−1^;
2: 3 min isotherm;
3: Heated until 35 °C, with a heating flow of 10 °C·min^−1^;
4: Cooled until 25 °C, with a cooling flow of 10 °C·min^−1^.

### 2.7. Scanning Electron Microscopy-Energy Dispersive Spectroscopy of X-rays (SEM-EDX)

#### 2.7.1. Substrate and Sample Preparation

A silicon substrate 500 µm thick, with a 4-inch size, was used for the measurements. A frame with several circular shapes was glued in the substrate, forming a pillar volume of 3.0 × 10^3^ µm × 120 µm (diameter × high). After gluing the frame, the substrate was pre-treated in an oven at 75 °C overnight.

The samples (resins, epoxidized resins, and Bisphenol-A) were diluted in cyclopentanone and spotted in the pillar volume shape. The substrate was heated until 95 °C overnight to evaporate the solvent.

#### 2.7.2. Parameters of the SEM-EDX

The measurements were made in the equipment model Zeiss Supra 60 VP, from Bruker Nano GmbH (Berlin, Germany). The parameters were 10 KeV for the beam; density: 6125; primary energy: 10 m; take off angle: 35; tilt angle: 0; azimut angle: 0; detector type: Bruker X-flash 6 series; detector thickness: 0.45; Si dead layer: 0.08; calibration, abs.: −477.65; Mn FWHM: 146.563; Fano factor: 0.145; channels: 2057; aperture: 120 µm; and current: 2.28 µmA.

## 3. Results and Discussion

### 3.1. Synthesis and Characterization of the Polymers/Oligomers

**Figure 2:**
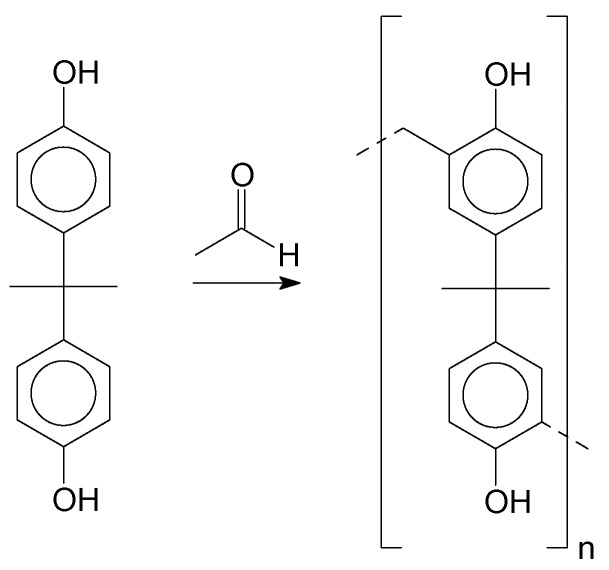
Polymerization of Bisphenol-A and formaldehyde.

The polymerization of the resins was performed using bisphenol-A as the monomer. Figure 2 depicts the polymerization reaction. After the synthesis procedure, the products were, in most cases, oligomers, with short chain sizes varying “n” from 1 to 5. 

**Figure 3:**
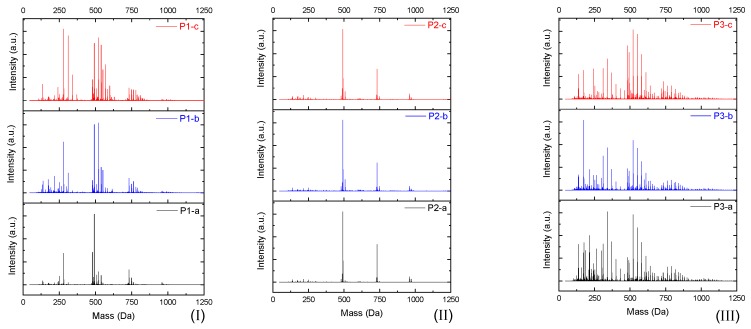
Electrospray Ionization-Time of Flight-Mass Spectroscopy (ESI-µ-TOF-MS) spectra of the triplicate reactions for each polymer profile. (**I**) P1, (**II**) P2, and (**III**) P3 reactions.

The products were characterized by ESI-µ-TOF-MS spectroscopy. The samples were evaluated initially in terms of the oligomer size, primary, and derivate of the primary products, following a similar methodology previously described in the work of Vlnieska et al. (2018) [1]. Three different polymers were synthetized and carefully studied here, named P1, P2 and P3. Figure 3 presents the ESI-µTOF-MS spectra, where triplicates of each polymer are compared.

Interestingly, the spectra show a distinct profile for each oligomer. The reaction P1 presents a mixture of monomers, dimers, and traces of trimers, with considerable amounts of derivate products (methoxyl radicals). The reaction P2 presents a clean profile, mainly composed of dimers and a low concentration of trimers. The reaction P3 presents the highest polydispersity, with secondary products in all oligomer compositions, monomers, dimers, trimers, and traces of tetramers. 

By adjusting the variables of the polymerization reaction, it is possible to obtain samples with low amounts of derivate products and samples with high amounts and spread the profile of derivate products, resulting in polymers with different mechanical properties.

**Figure 4:**
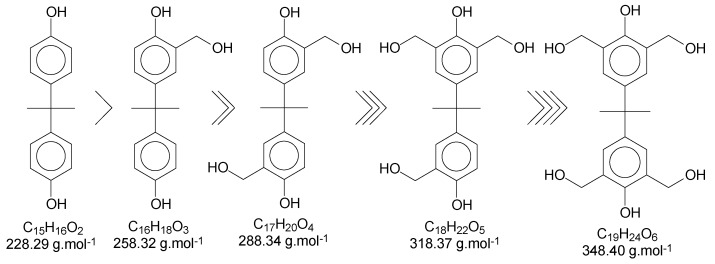
Derivate and intermediate products seen during the polymerization, considering the monomer as a “skeleton” example.

Once this polymerization reaction generates derivate products, it is not possible to achieve a “pure” polymer/oligomer, leading to the necessity to define what is considered as a “pure” polymer/oligomer, and what a derivate product is. For instance, the *mer* that has no other ramification besides the methylene connecting the monomers can be considered the “pure” oligomer; any other structures are then derivate products, which are variations in the end and in the reactive positions of the oligomer chains. Figure 4 represents a simplified version of the possibilities, without considering the reactions in all positions of the aryl ring and in the phenolic position, as well as representing the relative reactivity of each derivate/intermediate product.

**Figure 5:**
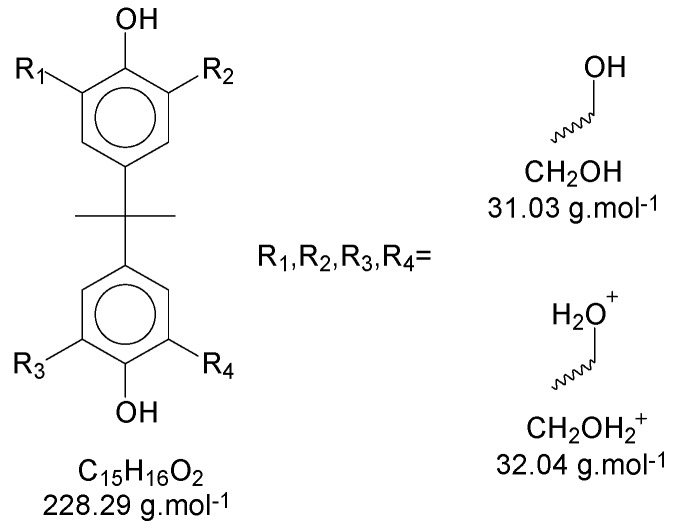
Proposed chemical structure for the termination groups.

Using Bisphenol-A as the basic structure and only the ortho positions at the aryl ring (the most reactive ones), for each monomer unit, it is possible to achieve four substitutions. When one of the substitutions is performed, the reactivity of this aryl ring tends to decrease once the substitution group is a moderate deactivator for the ring [9]. These derivate products are applicable to all chains of the polymer/oligomer, generating a wide range of possibilities related to the chain size. The longer the chain, the higher the chances are of obtaining more derivate products. The likelihood of viewing slight changes in the structure of the substitution groups might also be important to consider. Figure 5 presents the possible termination groups, taking into account that the positive mode was used for mass spectroscopy characterization.

**Figure 6:**
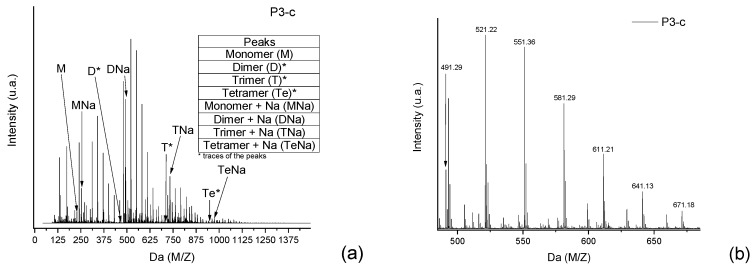
(**a**) Full spectra from the polymer P3, where the neutral *mers* and adducts with sodium are identified. (**b**) Secondary products in the dimer region of the P3 spectra.

Taking, as an example, the spectra from the reaction P3-c (Figure 2), the masses of each neutral *mer* from the polymer were selected (Figure 6a). Then, in the mass region of the dimer, most of the possibilities for the secondary products were selected (Figure 6b). The sodium adducts that can be formed during the ionization step might also be considered [12,13,14,15,16].

**Figure 7:**
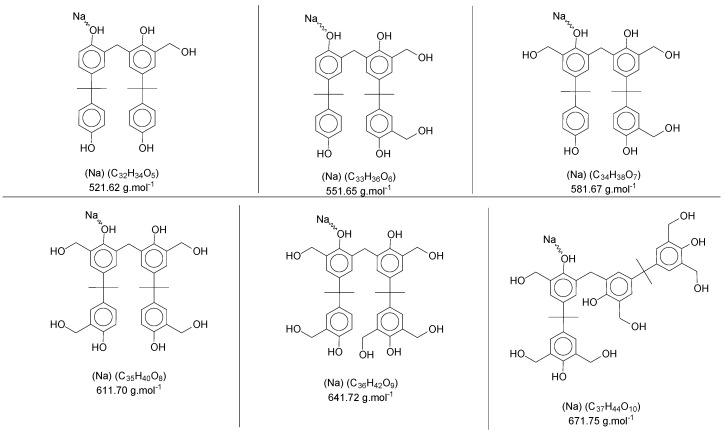
Sodium adducts from the secondary products in the dimer region′s spectra of the polymer P3.

In Figure 5a, the intensity of the peaks is higher—for all *mers*—with the sodium adducts. During the polymerization reaction, the addition of formaldehyde molecules is randomly distributed in the ortho positions of the aryl ring, generating the derivate products with intervals of approximately 30 Da (taking into account that these radicals can be in different structures, presented previously in Figure 4). Figure 7 shows the structures for the secondary products in the dimer region, with the sodium adduct possibilities.

The reactivity of the derivate products also seems to be reasonable with the proposal made in Figure 3, and in terms of the spectra, all secondary products for all monomers and all samples follow the same pattern with respect to the relative intensity, where the most intense peak represents the derivate product with one methoxyl group as ramification. The other peaks also follow a decreasing order of reactivity (Figure 6b as an example).

The same approach was applied to the other regions of Figure 6a, and the *mers* and secondary products were identified. Table S2 (supplementary material) presents the *mers* and their derivate products (mainly for P1 and P3) for each polymer, considering one or more sodium atoms as forming adducts and the number of radical groups inserted in the oligomer structure.

**Figure 8:**
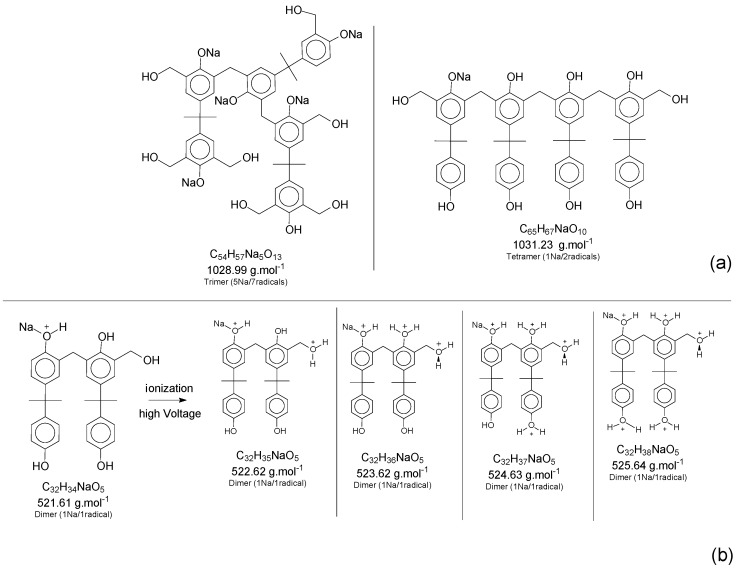
(**a**) Two different structures for the same range of mass. (**b**) Ionization of the phenol groups in one of the tetramers´ structure.

Although most of the peaks were identified with the theoretical values for adducts and derivate products, as presented in Figure 6, some values were in between two possibilities of structures, and even one single peak mass value can represent two distinct structures. This scenario can be explained by implications that should be considered for this characterization method, where, during the ionization step, the mass slightly changes the values, once the oligomers have several positions susceptible to being ionized (mainly the hydroxyl groups attached to the aromatic ring). To illustrate the concept, Figure 8 presents two situations: “a” is an example of two structures in the same range of theoretical mass and “b” represents the mass variations in the ionization step of the ESI-µTOF-MS spectroscopy in one of the tetramers identified. This assumption is only based on the ionization for the phenolic hydroxyl groups. 

Although the adducts with sodium, ionized structures, and the derivate products are randomly generated, leading to a broad spectrum of mass possibilities for the single structure, this characteristic helps to identify the molecules and prove the fingerprint of the reaction system applied. Figure 7 presents a specific mass peak from the structures in Figure 8b, including a derivate structure from the dimer oligomer, with one sodium atom and one radical. The variations are assigned for the ionization in the phenolic groups and the radical group.

**Figure 9:**
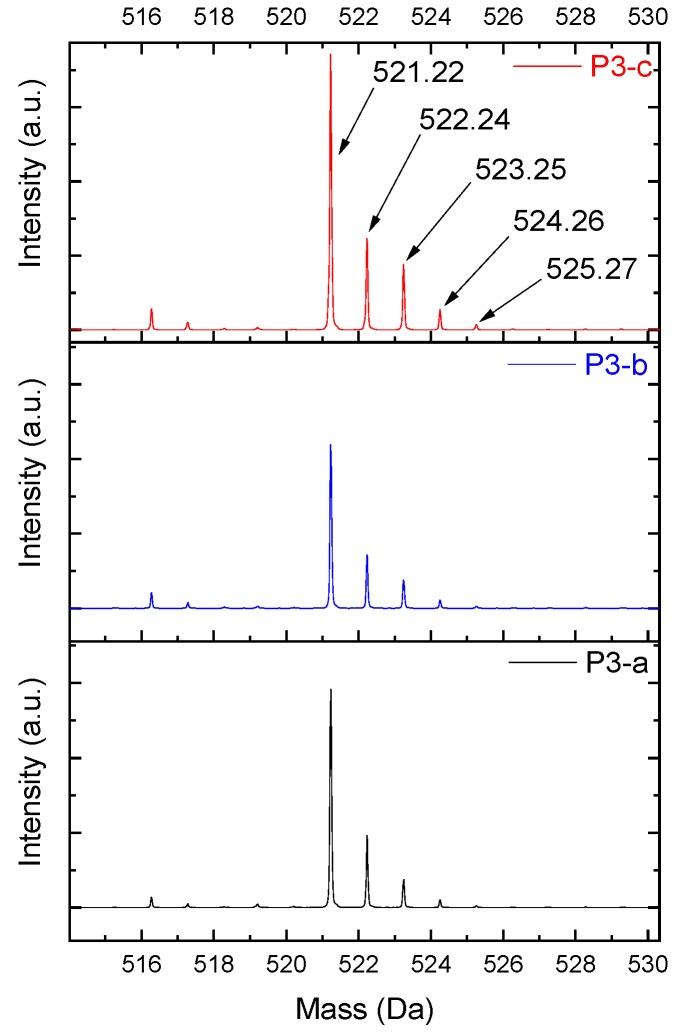
Dimer′s derivate (1 Na/1 rad.) spectra.

These variations in the Da mass were representatives for all intense peaks. The slight mass changes were present at lower intensities, at the boundaries of the intense peak, as Figure 9 presents. These attributions are suited to ionization in the phenolic positions, as well as in the radical groups identified.

**Figure 10:**
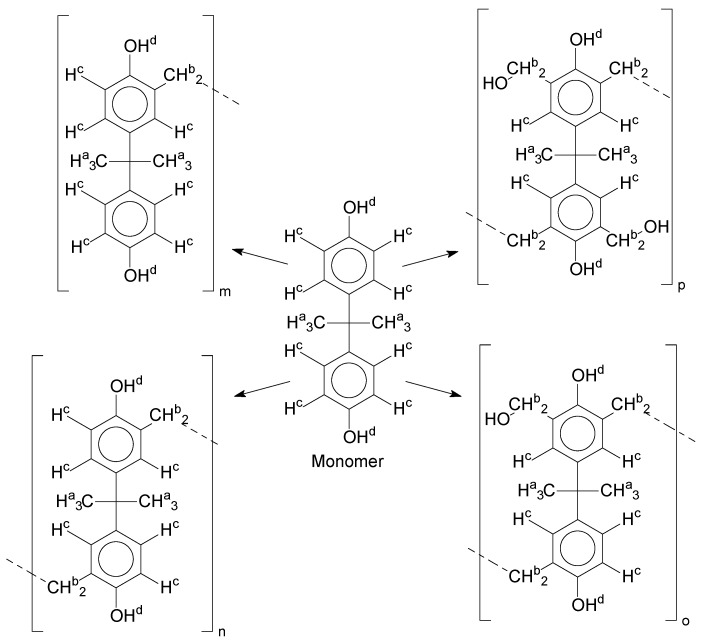
Monomer′s chemical structure and its possible derivate with labeled hydrogens.

Additionally, the oligomers were characterized by NMR spectroscopy. To gain a better understanding of the reactions, Figure 10 presents the chemical structure marked with the hydrogens of interest that were studied (based on the monomer structure), and Table 1 shows the amplified spectra regions and their integral values. The full spectra are available in the supplementary material (Figures S1–S4).

As previously explained, the derivates are expected to be mainly at the ortho positions of the aromatic ring. Derivates from meta positions, as well as the ones generated at the phenolic fractions, are not considered in this assumption.

The singlet at 2.83 ppm and the multiplet at 2.05 ppm are associated with solvent/moisture (see full spectra in the supplementary–Figures S1–S7). The multiplets in the region of 6.55–7.05 ppm are assigned to the aromatic hydrogens (“c”–Table 1). The deployment in the regions of phenolic aromatic groups (“d”–Table 1), methyl groups (“a”–Table 1), and phenolic groups (“d”–Table 1) are due to the polymerization reaction and its derivate, generating asymmetry in the structure. The comparison of region “a” and “d” shows that the phenolic groups are unreacted during the polymerization. 

The methylation of the resins can be calculated, as Equation 1 shows. The equation considers the comparison of the aromatic hydrogens area (8 hydrogens) and methyl hydrogens area (6 hydrogens). The Bisphenol-A spectrum was used as a reference value.

(1) $$s.r.=1-\left(\frac{c_{\mathrm{sample}}\times 2}{c_{\mathrm{bisphenolA}}}\;-\;1\right)$$

Here, s.r. is the substitution ratio and c is the integral value from the aromatic region (c–Table 1).

The integral of the aromatic region only provides information about the reacted amount of hydrogens, and it is not possible to distinguish if the product is a methylene bridge or a methyl-hydroxyl group. Table 2 presents the substitution ratio for each synthesized polymer.

Once the region “b” is assigned for the methylene bridges, methyl-hydroxyl groups, and possible residues of unreacted p-formaldehyde (one can see this for P3, where the signal in the range of 9.5 ppm confirms the p-formaldehyde traces), precise assignment for each group is not achievable [17].

The substitution ratio seems to agree with the ESI-µ-TOF-MS. A substitution ratio of 25% would be a methylene bridge/methyl-hydroxyl group for every monomer structure within the polymer chains. Considering that the polymer chains have “n” mostly in the range of 2 to 4, these values turn out to be a high substitution ratio, expressing a high content of derivative products for the oligomer P3, for example.

**Figure 11:**
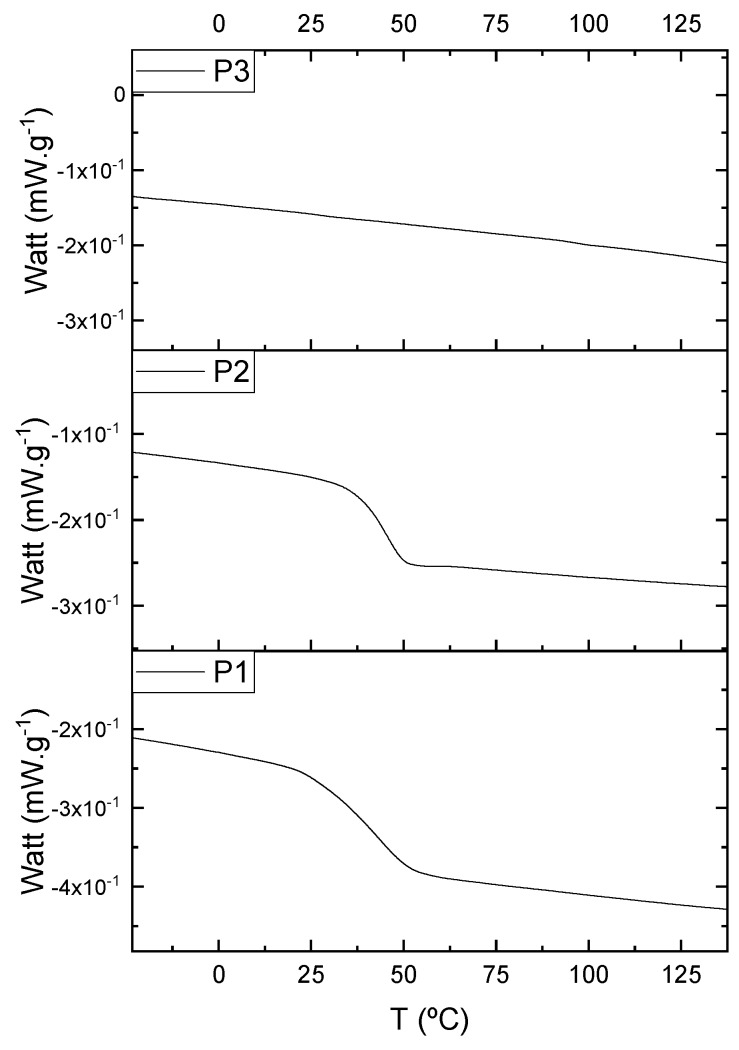
Differential scanning calorimetry (DSC) curves for the samples P1, P2, and P3.

The oligomers were evaluated by differential scanning calorimetry. Figure 11 presents the DSC graph for the samples P1, P2, and P3.

Comparing the samples, the DSC also shows three different profiles of heating behavior. The sample P1 presented *T*_g_ (transition glass temperature) starting at 30.7 °C, with 0.67 J·(g·°C)^−1^. The sample P2 exhibited *T*_g_ starting at 49.1 °C, with 0.71 J·(g·°C)^−1^. The sample P3 presented no *T*_g_, indicating that there is no crystalline phase present in this material. Regarding the samples P1 and P2, the crystalline phase is more pronounced for the sample P2 [18]. These results seem to indicate the following behavior: once the concentrations of the secondary products increase, the crystalline phases decrease. The DSC supports the results of the other characterization techniques, although the samples are all based on the same monomer, and each polymer presented a different structure, molecular size, and properties [19,20].

### 3.2. Alkylation of the Phenolic Groups and Characterization

**Figure 12:**
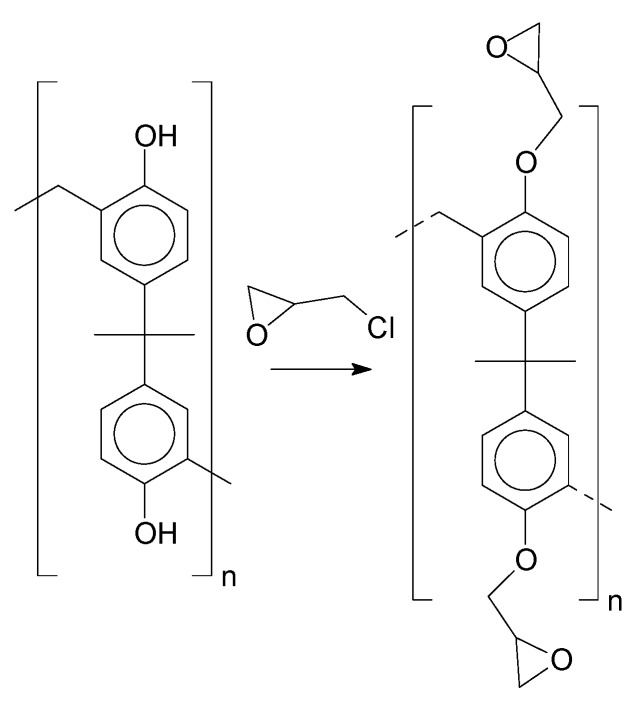
Alkylation reaction.

Insertion of the epoxy groups was conducted through the alkylation of the phenolic groups present in the structure of the resins. The synthesis was studied to promote the substitution of the phenolic groups as much as possible. Figure 12 shows the reaction proposed for this step.

**Figure 13:**
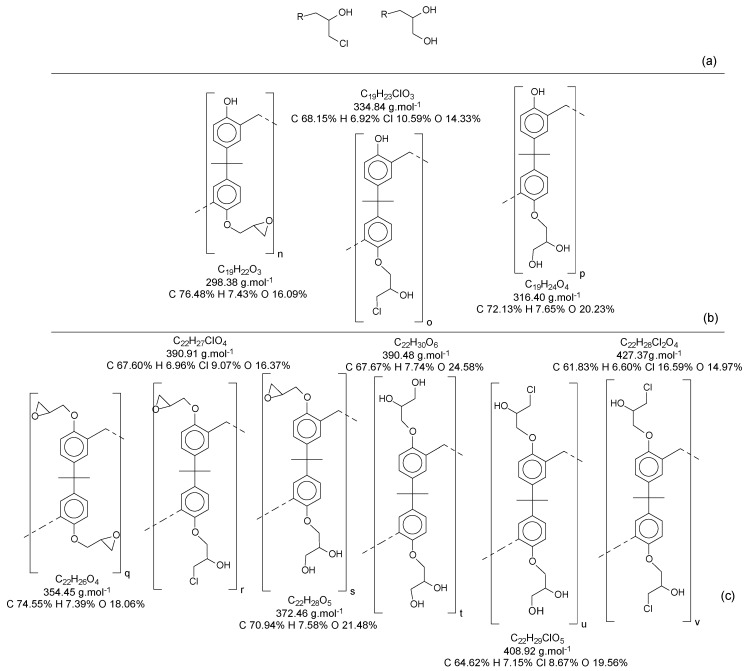
(**a**) Derivate from the epoxidation reaction; (**b**) Mono-epoxy derivate from the Bisphenol-A; (**c**) Di-epoxy derivate from the Bisphenol-A (epoxy weight = 2).

Figure 13 presents the possibilities of epoxy radicals and secondary products generated during the epoxidation reaction [21], using only the monomer as an example for the polymer chain. 

When all the oligomers obtained in the first reaction and their secondary derivate are considered, the possibilities for variations in structures and mass become increasingly wide. In this reaction, for each structure (as an example, the ones identified in Table S2), there is a possibility to generate at least three another derivate for each phenolic group. 

**Figure 14:**
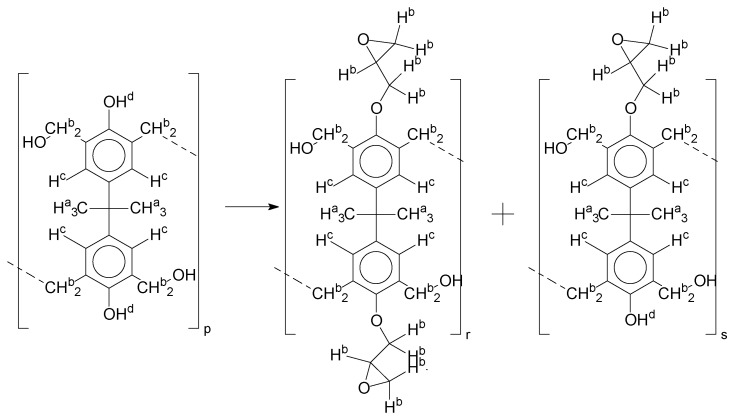
Oligomer “p” and epoxidized oligomer “r” and “s” chemical structures with labeled hydrogens.

As the epoxy groups are directly related to the photo-sensitivity of the photoresists, it is crucial to quantify the number of epoxy/derivate groups inserted at the oligomer chains. The characterization method applied for the quantification was NMR spectroscopy. The main peaks in the spectra were evaluated before and after the reaction. The methodology used to quantify the epoxy content was based on the work of Dorsey et al. (1977), Fleming (1985), and Garcia and Soares (2002) [22,23,24], with modifications to suit to this characterization. Table 2 presents the amplified spectra regions and their integral values, comparing the samples before and after the epoxidation reaction. The full spectra can be found in the supplementary material (Figures S5–S7). Figure 14 presents the chemical structure marked with the hydrogens of interest that were studied (based on the monomer structure).

For Figure 14, the oligomer chain with two hydroxyl groups in the monomer′s structure is considered as an example, and the partial substitution reaction, the oligomer “s”, is considered as a product.

The epoxy content could be determined by integration between the peaks assigned to the methyl groups from the polymeric chain and the region from 3.25 to 4.25 ppm, assigned to the hydrogens from the epoxy group [22,23,24,25]. However, for this kind of resin, the determination is not realistic; once the methyl hydroxyl groups from secondary products are also assigned to this ppm region, it is not possible to distinguish the signals among them. An alternative would be to use the phenolic signal, before and after the epoxidation reaction, taking into account that this value estimates only the reacted phenolic groups, representing the overall formation of the epoxy groups, with all possibilities (see Figure 13). Table 3 presents the ^1^H NMR for epoxy and non-epoxy resins. The regions shown were used to determine the epoxy content.

Table 4 shows the epoxidation ratios (e.r.) achieved for the samples P1-ep, P2-ep, and P3-ep.

**Figure 15:**
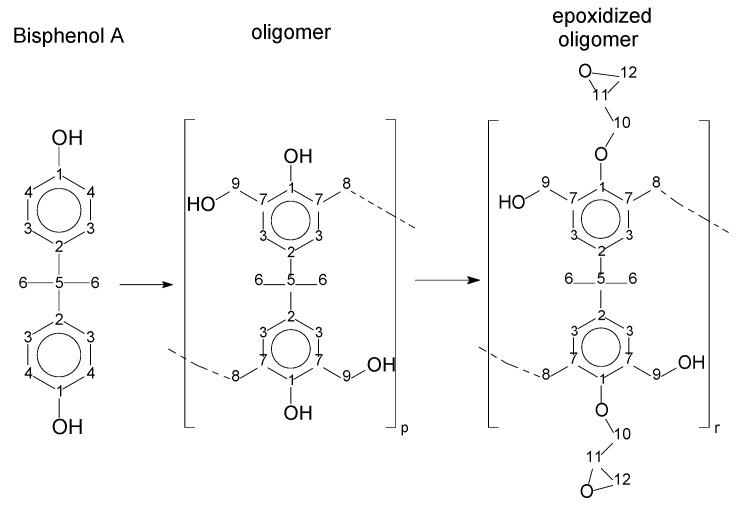
Chemical structures with numbered carbons.

The amounts of epoxy resins were also evaluated by the carbon experiment, based on the work of Fleming (1985) [23]. The carbon spectra can help to distinguish the epoxy and methylene groups. In this case, the presence of secondary epoxy products (Figure 13) does not significantly affect the shifting signal. Figure 15 presents the chemical structures with numbered carbons. Table 5 presents the ppm values assigned for each carbon, for the Bisphenol-A, the oligomers, and their epoxidized products. The full spectra are presented in the supplementary material (Figures S8–S14).

The multiplet at approximately 30 ppm is associated with the solvent, as well as the peak at 206 ppm. The peaks from 27 to 31 ppm represent the methyl groups and the methylene bridges (6 and 8–Figure 15). The peaks from 41 to 51 ppm are associated with the quaternary carbon (5–Figure 15). The region between 45 and 75 ppm is assigned to the epoxy group carbons (10, 11 and 12–Figure 15), except for the peak at 63 ppm, which is assigned to the carbon´s methylene bridge (8–Figure 15). The peaks related to the aromatic carbons are in the region near to 115 ppm (meta) and 130 ppm (ortho) (3 and 4–Figure 15). The peak near 140 ppm is associated with the carbons at the para position (2–Figure 15). The peak near 160 ppm is related to the carbon bound in the hydroxyl group (1–Figure 15). Comparing the bisphenol-A and the resins P1, P2, and P3, one can see a significant asymmetry, as evidenced by the deployment of the peaks in the spectra (see Table 4 and number of deployed peaks). This is due to the polymerization reaction. The presence of epoxy carbons in the region from 50 to 75 ppm, which is evident from the comparison of resins and their epoxidized products, confirms the reaction quantified by proton NMR.

The amounts of epoxy groups were also compared using SEM-EDX. Table 6 presents the relative ratios of the atoms of samples P1 to P3 and P1-ep to P3-ep. The spectra from the samples can be found in the supplementary material, in Figure S15.

Table 6 presents the average of triplicates (the full data can be found in Table S3, in the supplementary material). Considering the structure of the resin purely as an oligomer, without ending groups and derivates, the theoretical value of the relative percentage of oxygen would be 13.2%. The resins showed an average of 15.2% of oxygen atoms, suggesting that circa 2.0% of the oxygen atoms come from termination groups and/or derivates, as presented in Figure 4 and Figure 5. The small amounts of chlorine in the resins are due to slight contamination from the epoxidized resins over time during exposure to the high voltage in the SEM, since the samples were processed together.

Regarding the epoxidized samples, an average increase of 4.6% for the oxygen atoms was observed, as well as 5.4% for the chlorine atoms. 

In order to interpret this reaction system with a better accuracy, some boundary conditions were applied:

1: Once the alkylation was performed in excess of epichlorohydrin, it would be possible to expect alkylation via epoxy groups, increasing the epoxy weight [22,23,24,26,27];
2: To evaluate the derivate from the alkylation reaction, we considered for elucidation only the possibilities from one to five epoxy groups by each bisphenol molecule, following the respective derivate from the epoxy groups (Figure 13 and Figure S16).

An increase of oxygen in the samples is expected and indicates the alkylation reaction, while the chlorine amount indicates an interesting achievement, and the epoxy groups could be composed in reality by most of the chlorine′s derivate (Figure 13a).

The compositions of the elemental analysis indicate that the epoxy resins have structures varying from 1:3 to 1:4 (BisphenolA:epichlorohydrin). Epoxy weights higher than these proportions were disregarded, since the percentage of oxygen atoms is in the range of 30%. The chlorine atoms percentage of 5.4% indicates the chloride version of the epoxy groups, containing only one chlorine atom per epoxidized *mer*, as presented in Figure S16, structure numbers 3, 5, 9 and 11.

**Figure 16:**
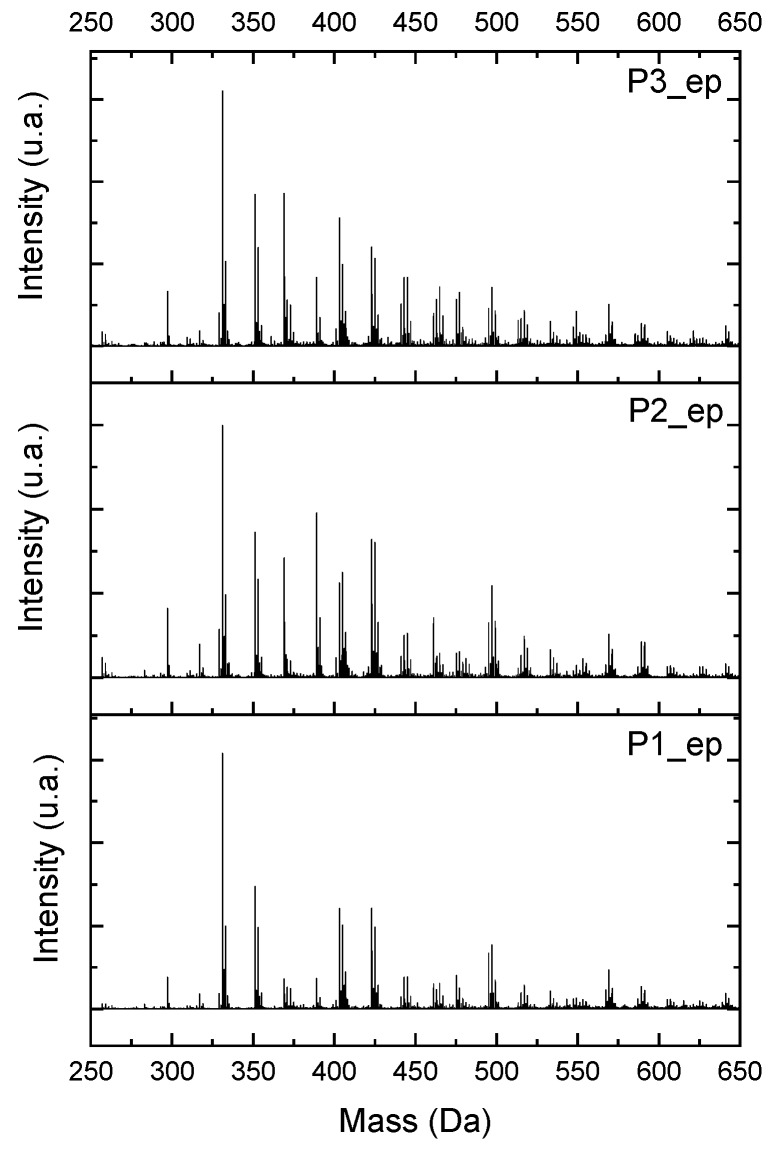
Electrospray Ionization-Time of Flight-Mass Spectroscopy (ESI-µ-TOF-MS) spectra obtained from epoxidized resins, amplified in the monomer region.

Using ESI-µ-TOF-MS, we considered the region of the monomer for this characterization. Once the chain of the *mers* grows, there is a possibility of geometric derivate progress, and it is almost impossible to characterize each single derivate from the *mers* in the composition of the resins. Figure 16 presents the mass spectra, in the region of the monomer for the samples P1-ep, P2-ep, and P3-ep. The full spectra can be found in Figure S17 in the supplementary material.

Following the approach used to characterize the oligomers from the resins (Figure 5, Figure 6, Figure 7, Figure 8 and Figure 9), observing the mass spectra in Figure 16, all the structures from Figure 13 and Figure S16 can be present in the composition of the epoxidized resins; however, the concentrations of the compounds were higher in the region of 1:2 to 1:3 (Bisphenol:Epichlorohidrin), with masses from 340 to 505 g·mol^−1^, presented by the structures in Figure 13c and Figure S16 (structures 3, 5, 9 and 11).

The hydrogen NMR also indicates a ratio between 1:2 and 1:3 (Bisphenol:Epichlorohidrin) for the epoxidized resins. To elucidate this ratio, the integral values assigned for the epoxy groups were compared with the integral values assigned for the methyl groups from Bisphenol-A. To have a more accurate measure, were prepared ten experimental measures, using Bisphenol-A and Epichlorohidryn as standards, varying the ratio from 1:1 to 1:10. Table 7 presents the integral values, and the full spectra can be found in supplementary material, Figure S18.

The substitution ratio for the epoxidized resins seems to be in accordance with the results for the elemental analysis and the ESI-µ-TOF-MS, once P1-ep, P2-ep, and P3-ep present integral values from the epoxy groups of 1.76, 1.80 and 1.86, respectively. These results confirm the probable mixture of oligomers composed by a 1:2 and 1:3 ratio of epoxidation.

**Figure 17:**
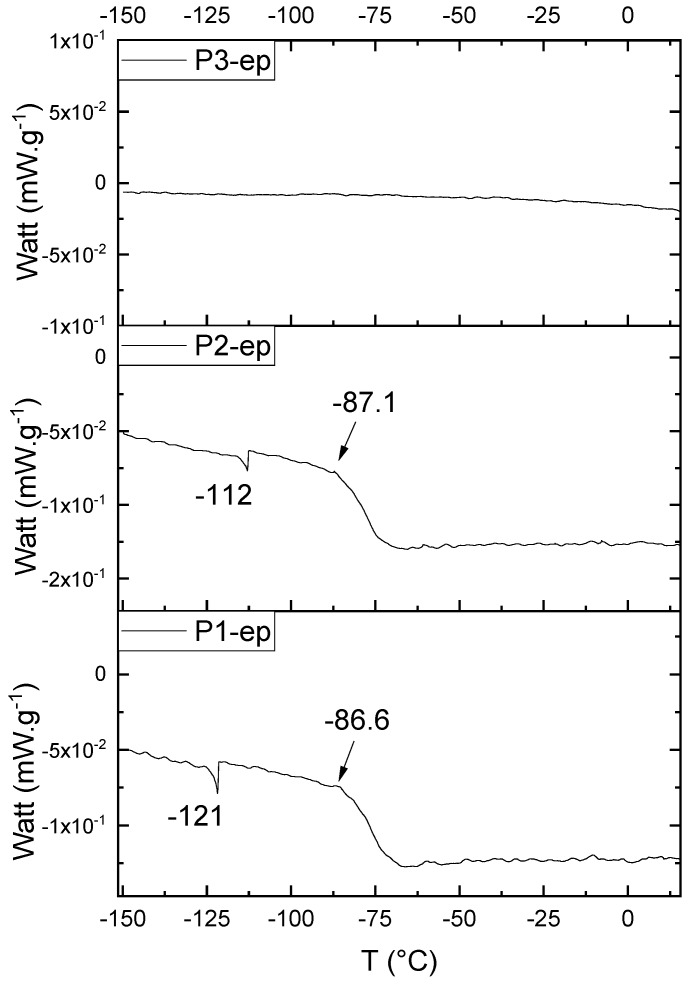
Differential Scan Calorimetry (DSC) curves of the epoxidized samples P1-ep, P2-ep, and P3-ep.

Figure 17 presents the thermal behavior of the epoxidized resins.

Comparing the samples before and after the epoxidation reaction (Figure 11 and Figure 17), the profiles of the products are overall the same. However, the epoxidized samples presented *T*_g_ at a low temperature (reduced to approximately 110 °C, when comparing the resins P1;P1-ep and P2; P2-ep). The samples P1-ep and P2-ep also presented a *T*_g_′, assigned for small free movements at the end of the polymeric chains [18,19,20]. The sample P1-ep presented *T*_g_ starting at −86.6 °C, with 0.77 J·(g·°C)^−1^. The sample P2-ep presented *T*_g_ starting at −87.1 °C, with 0.70 J·(g·°C)^−1^. The sample P3-ep still shows a profile without *T*_g_ or *T*_g_′. After the epoxidation reaction, the P1 and P2 samples seem to present a similar crystalline phase, once the enthalpy values are close and the slopes of the derivative curve are similar [19]. The *T*_g_′ for P1-ep is 9 °C lower, indicating that the polymer chains have little more freedom to move. These achievements still show the following behavior: once the concentrations of the secondary products increase, the crystalline phase decreases.

## 4. Conclusions and Outgoings

Epoxy resins were synthesized, characterized, and tuned in this work, regarding the deep level of properties. They were synthesized using Bisphenol-A as a monomer, followed by an alkylation reaction. Three different profiles of polymer chains were achieved. The products presented a wide range of secondary products and their derivates, which were identified by mass spectroscopy (ESI-µ-TOF-MS), in accordance with NMR and elemental analysis (SEM-EDX). The resins were mostly composed of a mixture of monomers, dimers, trimers, and traces of tetramers. The epoxy groups were characterized and quantified, presenting an alkylation ratio near to 96% in the phenolic positions. The alkylation reaction also presented a derivate, and a good estimation about the structures and composition could be produced, indicating an epoxidation ratio between 1:2 and 1:3 (BisphenolA:Epichlorohydrin), with the chloride derivate in the epoxy groups.. The DSC measurement confirmed the unique profile for each batch of polymer, where the enthalpies of the glass transition were significantly distinct before and after the alkylation reaction.

The results lead us to conclude the following:

1: It is essential to define a profile of epoxy resin to be produced;
2: The final application should define the profile of the resin, e.g., DUVL, DXRL, et al.;
3: It is fundamental to strictly control the parameters of the reaction to achieve the desired profile of the resin.

## Figures and Tables

**Table 1:** Proton (^1^H) Nuclear Magnetic Resonance Spectroscopy (NMR) spectra (zoom in) for the samples Bisphenol-A, P1, P2, and P3.

Hydrogens (Figure 10)		d	c	b	a
**Sample**	**Bisphenol-A**	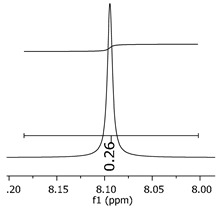	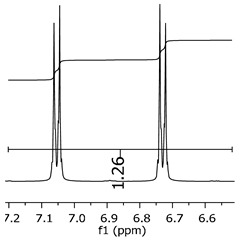	No peaks	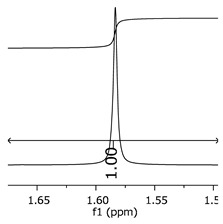
	**P1**	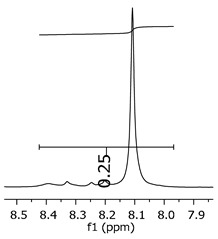	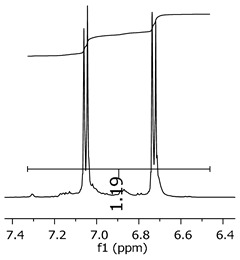	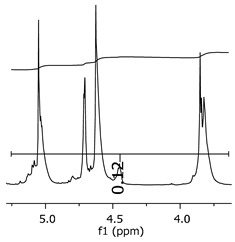	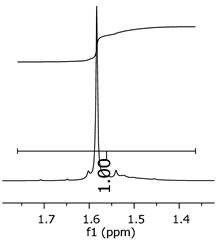
	**P2**	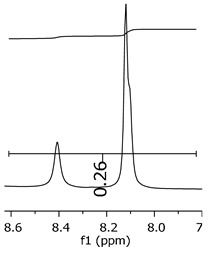	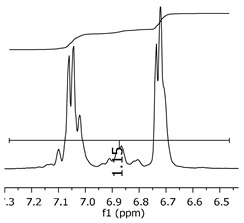	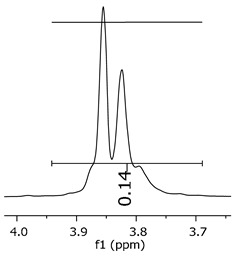	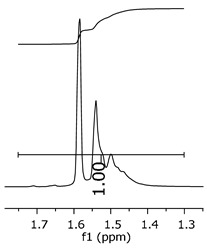
	**P3**	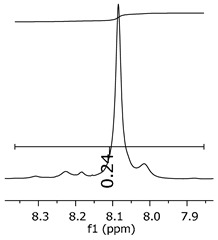	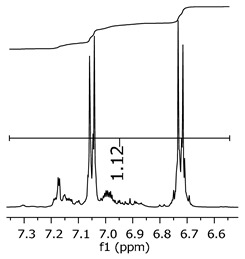	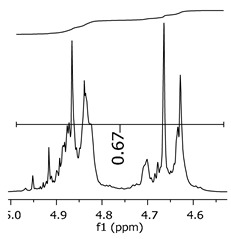	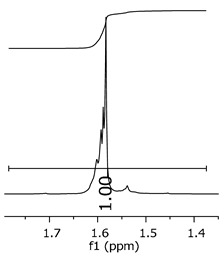

**Table 2:** Substitution ratio for polymers P1, P2, and P3.

Sample	s.r. (%)
P1	11.2
P2	17.5
P3	22.3

**Table 3:** Proton (^1^H) Nuclear Magnetic Resonance Spectroscopy (NMR) spectra (zoom in) for the samples P1, P1-ep; P2, P2-ep; and P3, P3-ep.

Hydrogens (Figure 10)		c	b	a
**Samples**	**P1**	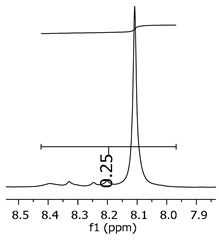	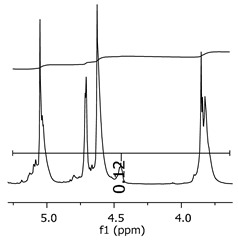	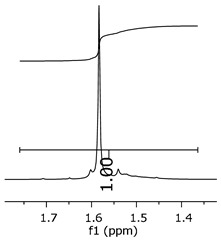
	**P1-ep**	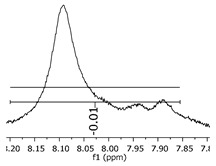	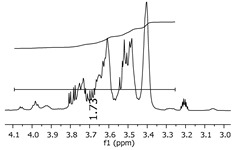	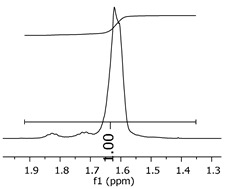
	**P2**	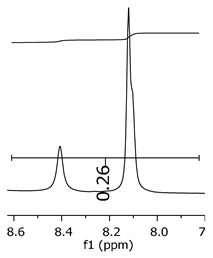	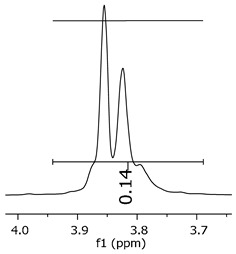	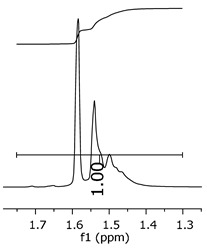
	**P2-ep**	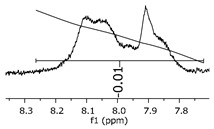	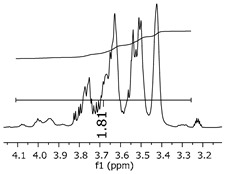	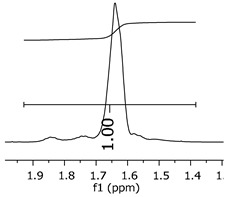
	**P3**	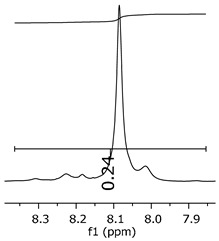	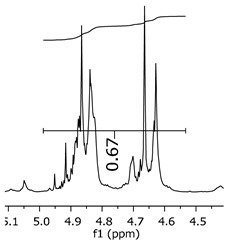	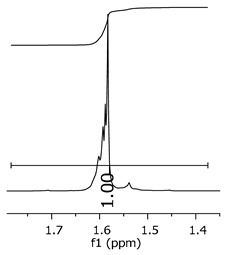
	**P3-ep**	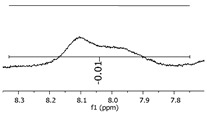	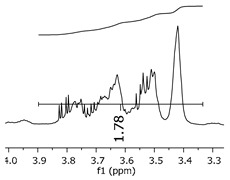	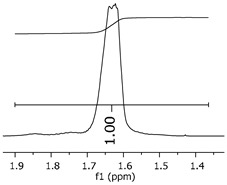

**Table 4:** Epoxidation ratios for the samples P1-ep, P2-ep, and P3-ep.

Sample	e.r. (%)
P1-ep	96.00
P2-ep	96.15
P3-ep	95.83

**Table 5:** Carbon (^13^C) Nuclear Magnetic Resonance Spectroscopy (NMR) shift values (ppm) for the samples Bisphenol-A, P1, P2, P3, P1-ep, P2-ep, and P3-ep.

Carbons (Figure 14)		1	2	3	4	5	6	7	8	9	10	11	12
**Samples**	**Bisphenol-A**	155 (1)	142 (1)	115 (1)	128 (1)	42 (1)	31 (1)	-	-	-	-	-	-
	**P1**	156 (6)	142 (2)	115 (2)	-	42 (2)	31 (1)	128 (5)	-	-	-	-	-
	**P1-EP**	157 (1)	143 (1)	114 (4)	-	45 (2)	27 (10)	128 (1)	27 (10)	68 (1)	71 (9)	51 (2)	46 (4)
	**P2**	152 (3)	143 (6)	115 (2)	-	42 (1)	31 (3)	128 (10)	-	-	-	-	-
	**P2-EP**	157 (1)	143 (1)	114 (1)	-	43 (6)	27 (9)	128 (1)	27 (9)	68 (2)	71 (12)	51 (2)	46 (4)
	**P3**	154 (3)	142 (2)	115 (5)	-	42 (3)	31 (3)	126 (8)	-	-	-	-	-
	**P3-EP**	-	-	114 (1)	-	44 (3)	27 (8)	128 (1)	27 (8)	68 (2)	71 (10)	51 (2)	46 (4)

Legend: AAA (B)–AAA = mean ppm value; (B) = number of deployed peaks in the ppm mean region.

**Table 6:** Relative atoms percentage ratio for the non-epoxidized and epoxidized resins.

Sample	Relative% of Atoms			
	Carbon	Oxygen	Chlorine	Silicon
P1	84.27 (±8.0)	15.72 (±2.6)	-	-
P1-ep	75.77 (±8.7)	19.09 (±2.9)	5.12 (±0.97)	-
P2	84.90 (±8.2)	14.83 (±2.5)	0.39 (±0.09)	-
P2-ep	75.01 (±8.7)	19.17 (±2.9)	5.63 (±1.1)	0.26 (±0.04)
P3	84.64 (±8.2)	15.02 (±2.5)	0.34 (±0.07)	-
P3-ep	73.77 (±8.5)	21.12 (±3.0)	5.46 (±1.0)	0.64 (±0.10)

**Table 7:** Integral values for epoxy groups versus the molar ratio.

Ratio ^a^	Integral ^b^
1:1	0.78
1:2	1.51
1:3	2.24
1:4	2.80
1:5	3.77
1:6	4.42
1:7	5.07
1:8	5.89
1:9	6.60
1:10	7.26

^a^ Ratio of Bisphenol-A:Epichlorohydrin. ^b^ Integral values assigned for epoxy groups.

## Supplementary Materials

The following are available online at https://www.mdpi.com/2073-4360/11/9/1457/s1.
