# Supplementary material for: Epoxy Resins for Negative Tone Photoresists
Source: Polymers (Basel). 2019 Sep 6;11(9):1457. doi: 10.3390/polym11091457 (PMC6780111; doi:10.3390/polym11091457)
Supplement: Supplementary file 1 [file polymers-11-01457-s001.pdf]

Supplementary Material

Table S1: Evaluated range of values for the parameters in the polymerization reaction.

**Experiment**	**Temperature** (°C)	**Solvent Molar ratio** (bisphenol-A: H_2O)	**Molar ratio** (bisphenol-A:formaldehyde)	**Time** [a] (hours)
01	130	1:31.68	1:0.5	0.5
02	130	1:31.68	1:0.6	0.5
03	130	1:31.68	1:0.7	0.5
04	130	1:31.68	1:0.8	0.5
05	130	1:31.68	1:0.9	0.5
06	130	1:31.68	1:1	0.5
07	60	1:31.68	1:1.5	0.01 to 8
08	75	1:31.68	1:3.0	0.01 to 8
09	90	1:31.68	1:5.0	0.01 to 8
10	100	1:31.68	1:1.5	0.01 to 8
11	115	1:31.68	1:3.0	0.01 to 8
12	130	1:31.68	1:5.0	0.01 to 8
13	120	1:31.68	1:1.5	0.01 to 8
14	135	1:31.68	1:3.0	0.01 to 8
15	150	1:31.68	1:5.0	0.01 to 8
16	150	1:31.68	1:1.5	0.01 to 8
17	165	1:31.68	1:3.0	0.01 to 8
18	180	1:31.68	1:5.0	0.01 to 8
19	60	-	1:1.5	0.01 to 8
20	75	-	1:3.0	0.01 to 8
21	90	-	1:5.0	0.01 to 8
22	100	-	1:1.5	0.01 to 8
23	115	-	1:3.0	0.01 to 8
24	130	-	1:5.0	0.01 to 8
25	120	-	1:1.5	0.01 to 8
26	135	-	1:3.0	0.01 to 8
27	150	-	1:5.0	0.01 to 8
28	150	-	1:1.5	0.01 to 8
29	165	-	1:3.0	0.01 to 8
30	180	-	1:5.0	0.01 to 8

[a] each reaction (7 to 30) was evaluated in intervals of 10 min approximately, collecting aliquots of the reaction.

Table S2: Characterization of the mers and the secondary products for the polymers VV22, VV25, and VV27, based on the spectra presented in Figure 3.

Polymer	Mer	Adduct/radical[a]	Mass (Da)[b]	Relative Intensity[c,e] (monomer level)	Relative intensity[d,e]
P1	Monomer	Adduct	-	-	**
		1Na	250.99	*	
		Sec. Product	-	-	
		1Na/rad	280.88	***	
		1Na/2rad	311.02	***	
		1Na/3rad	341.14	**	
		1Na/4rad	375.17	Trace	
	Dimer	Adduct	-	-	***
		1Na	491.22	***	
		3Na	537.48	***	
		Sec. Products	-	-	
		1Na/1rad	521.40	***	
		1Na/2rad	551.44	**	
		3Na/1rad	568.97	***	
		1Na/3rad.	581.26	*	
		3Na/2rad.	598.99	**	
	Trimer	Adduct	-	-	*
		1Na	731.31	***	
		2Na	749.67	***	
		4Na	797.49	**	
		6Na	839.41	*	
		Sec. Product	-	-	
		1Na/1rad.	761.02	***	
		2Na/1rad.	779.24	***	
		1Na/2rad.	791.37	**	
		2Na/2rad.	809.72	**	
		1Na/3rad. or 5Na	820.94	*	
		4Na/1rad.	827.06	*	
		1Na/4rad. or 5Na/1rad.	851.54	*	
		2Na/7rad. or 3Na/6rad	959.07	*	
		1Na/8rad.	971.19	*	
		4Na/6rad.	989.89	*	
		5Na/6rad.	1001.11	*	
		6Na/6rad.	1019.80	*	
P2	Monomer	Adduct	-	-	*
		1Na	251.13	*	
	Dimer	Adduct	-	-	***
		1Na	491.21	***	
		Sec. Product	-	-	
	Trimer	Adduct	-	-	**
		1Na	731.39	***	
		2Na	747.36	*	
		Sec. Product	-	-	
		2Na/7rad. or 3Na/6rad.	959.44	*	
		5Na/5rad.	972.45	*	
P3	Monomer	Adduct	-	-	**
		1Na	253.62	*	
		Sec. Product	-	-	
		1Na/2rad.	312.15	***	
		1Na/3rad.	341.41	***	
		1Na/4rad.	370.68	***	
	Dimer	Adduct	-	-	***
		1Na	491.29	***	
		Sec. Product	-	-	

		1Na/1rad.	521.22	***	
		1Na/2rad.	551.36	***	
		1Na/3rad.	581.29	***	
		1Na/4rad.	611.21	**	
		1Na/5rad.	641.13	**	
		1Na/6rad.	671.18	*	
		3Na/6rad.	721.49	**	
		4Na/6rad.	733.02	***	
	Trimer	Adduct	-	-	**
		1Na	733.02	***	
		5Na	821.68	**	
		Sec. Product	-	-	
		1Na/1rad.	761.42	***	
		1Na/1rad.	791.55	***	
		2Na/2rad. or 3Na/1rad.	809.23	**	
		1Na/3rad.	821.68	**	
		2Na/3rad	839.46	**	
		1Na/4rad.	851.39	**	
		2Na/4rad. or 3Na/3rad.	869.40	*	
		6Na/2rad	899.44	*	
		1Na/6rad.	911.43	Trace	
		6Na/3rad.	929.47	Trace	
		1Na/7rad. or 5Na/4rad.	941.36	Trace	
		2Na/7rad. or 6Na/4rad.	961.49	Trace	
		1Na/8rad.	973.30	Trace	
		6Na/5rad.	989.54	Trace	
		5Na/6rad.	1001.57	Trace	
		6Na/6Rad.	1019.57	Trace	
		5Na/7rad.	1031.59	Trace	
		6Na/7rad.	1049.62	Trace	
		5Na/8rad.	1061.61	Trace	
		6Na/8rad.	1079.61	Trace	
	Tetramer	Adduct	-	-	*
		1Na	973.30	Trace	
		2Na	989.54	Trace	
		5Na	1061.61	Trace	
		Sec. Product	-	-	
		1Na/1rad.	1001.57	Trace	
		2Na/1rad.	1019.57	Trace	
		1Na/2rad.	1031.59	Trace	
		2Na/2rad. or 3 Na/1rad.	1049.62	Trace	
		1Na/3rad.	1061.61	Trace	
		6Na	1079.61	Trace	
		1Na/4rad.	1091.63	Trace	
		6Na/1rad.	1109.67	Trace	
		1Na/5rad.	1121.65	Trace	
		6Na/2rad.	1139.37	Trace	

[a] Number of sodium atoms and radicals composing the adduct/ramified mer

[b] Dalton mass for adducts with one or more sodium atoms or secondary products with one or more sodium and/or radicals in the mer chain

[c] Relative intensity - Monomer level: Qualitative Intensity comparison within the mer spectra region

[d] Relative intensity: Mers intensity comparison in the overall spectra

[e] Qualitative rating: * - minor concentration; ** - approxymately middle concentration; *** - predominant concentration

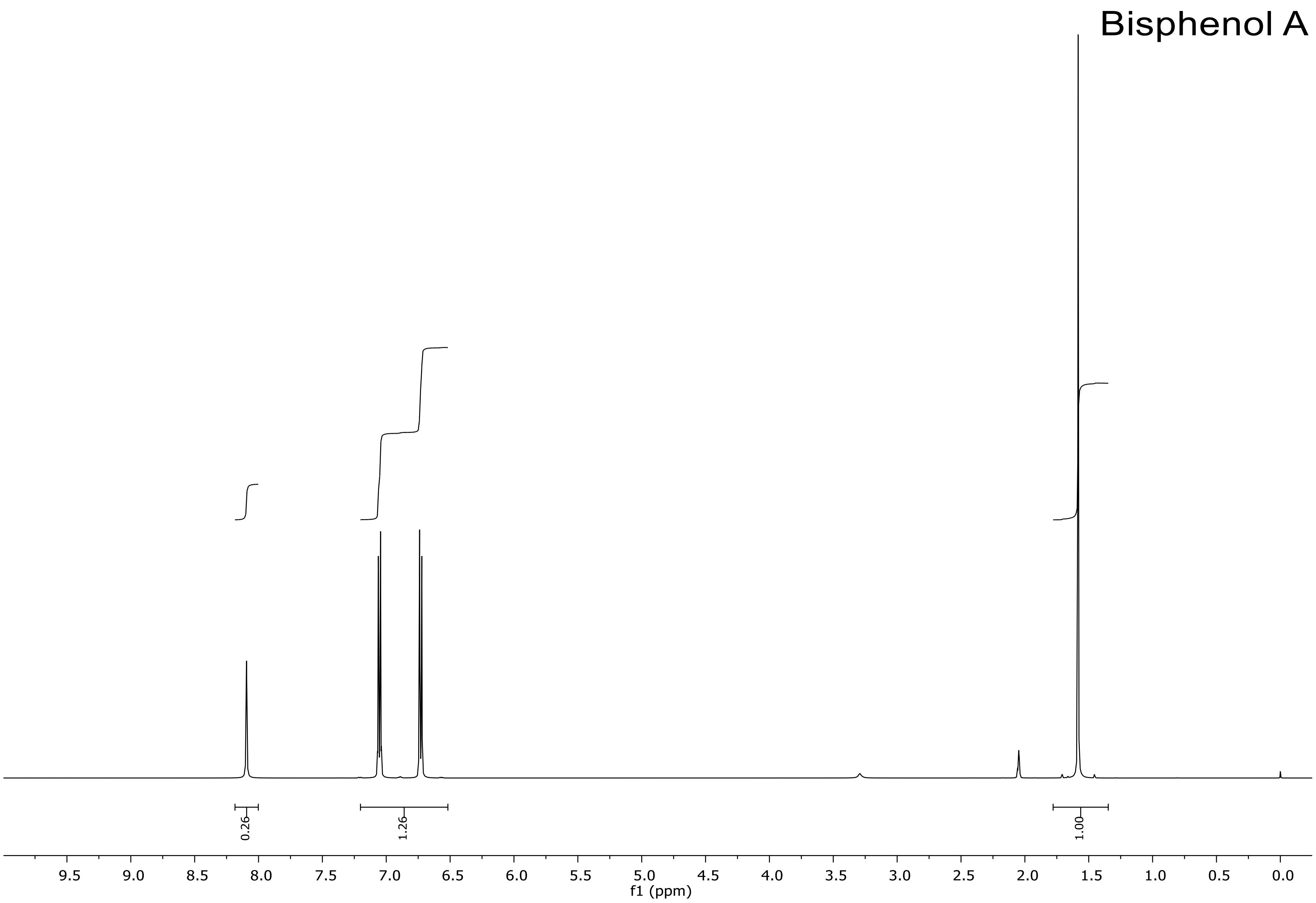

Figure S1. Bisphenol-A proton NMR.

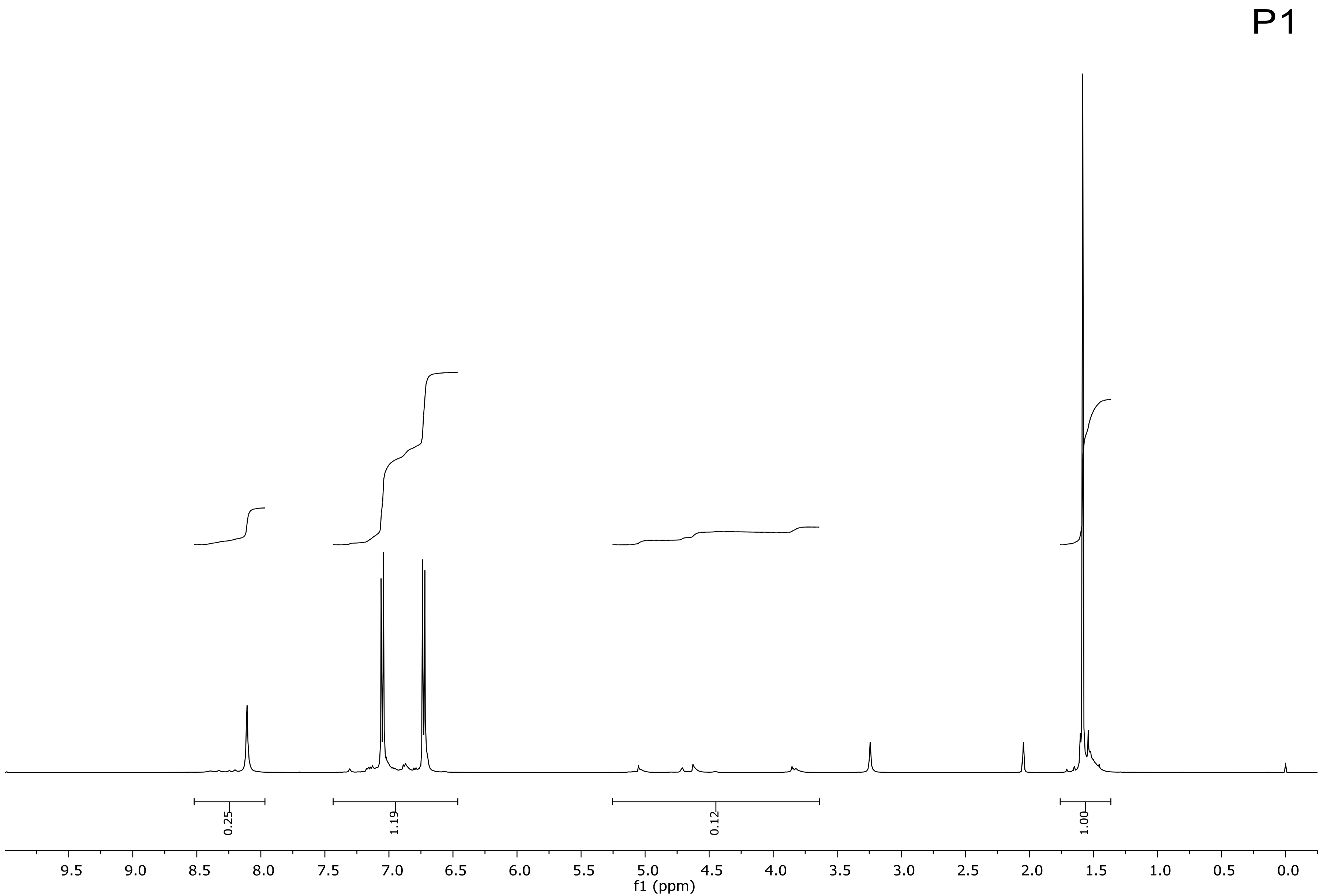

Figure S2. P1 proton NMR.

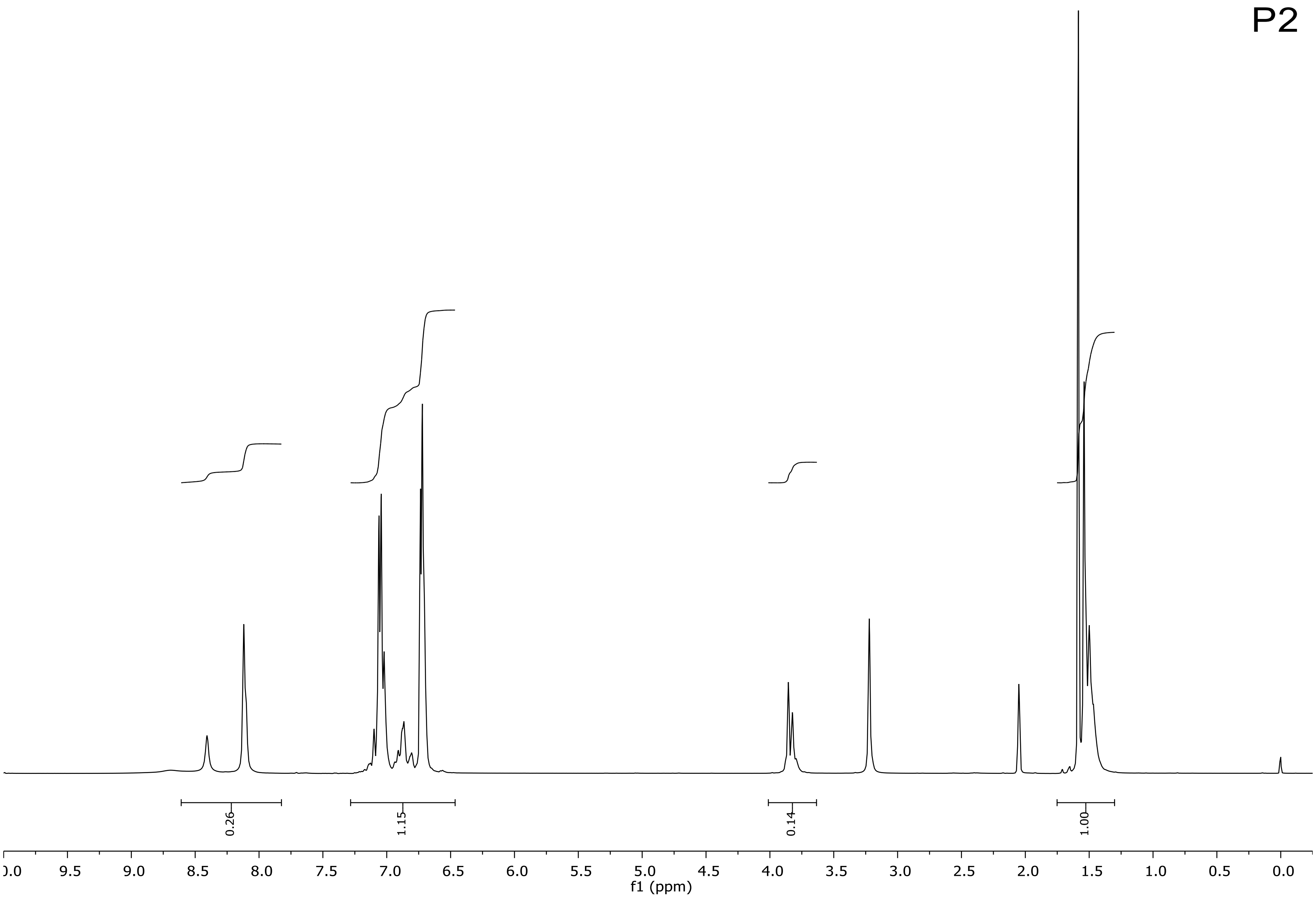

Figure S3. P2 proton NMR.

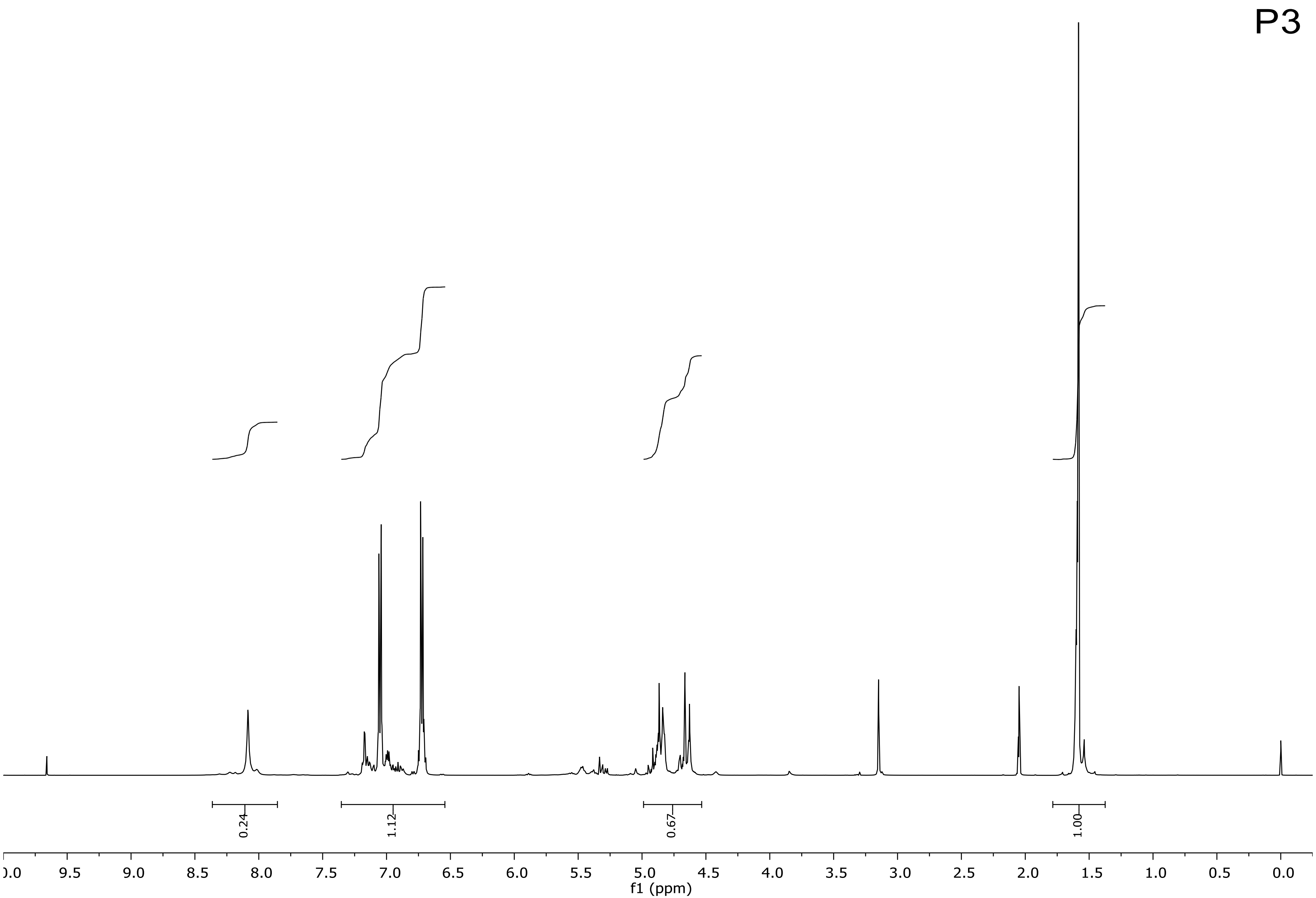

Figure S4. P3 proton NMR.

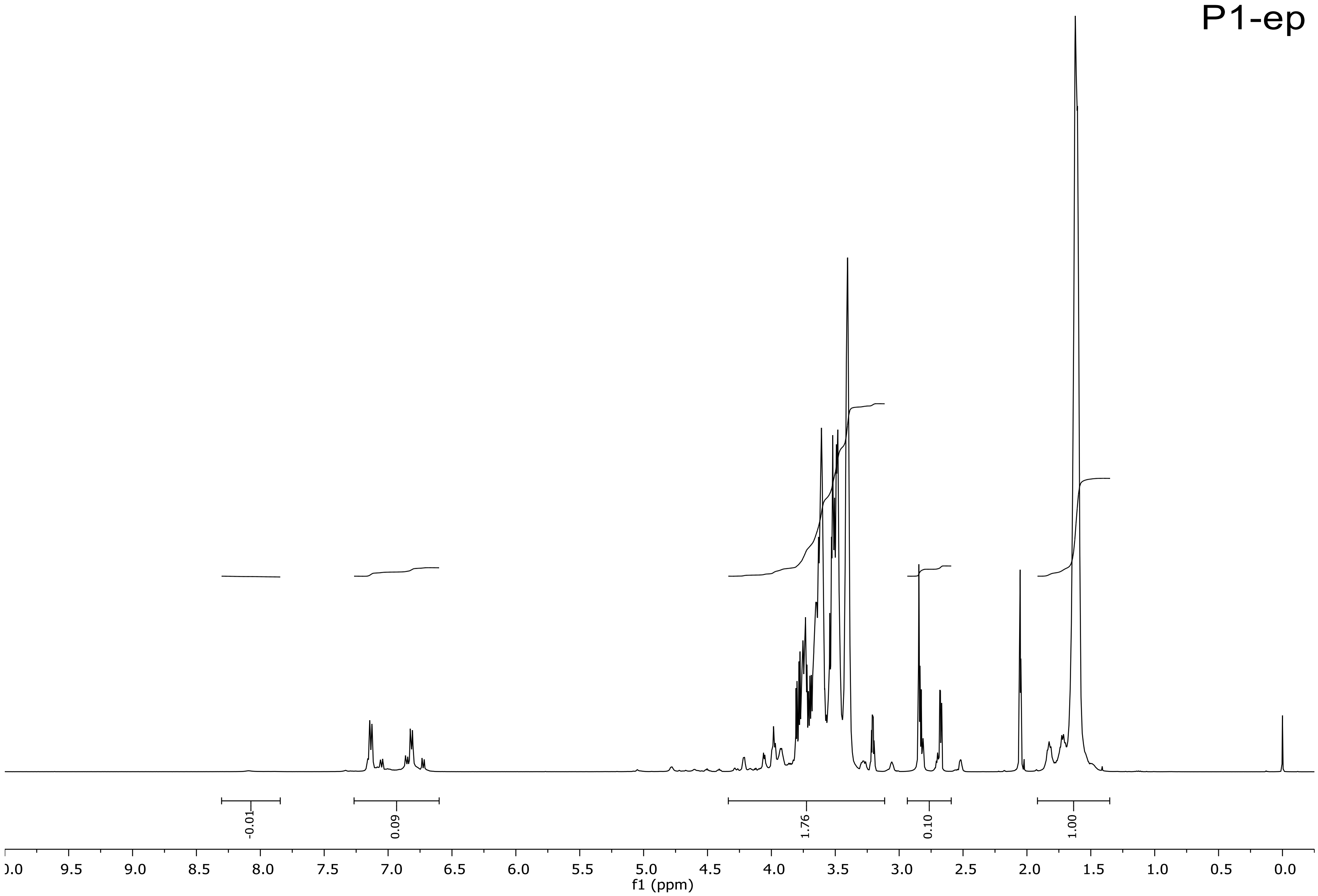

Figure S5. P1 epoxidized proton NMR.

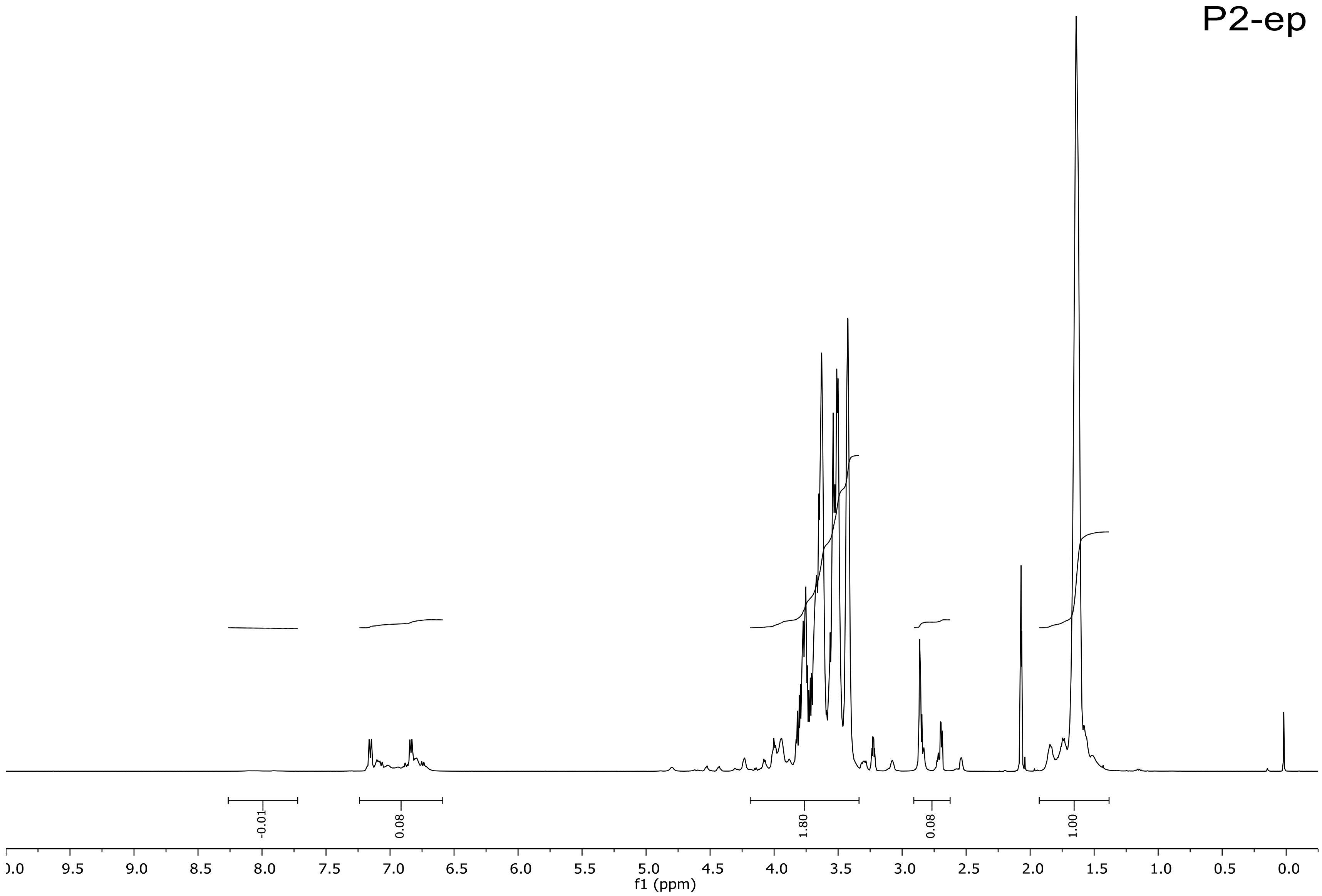

Figure S6. P2 epoxidized proton NMR.

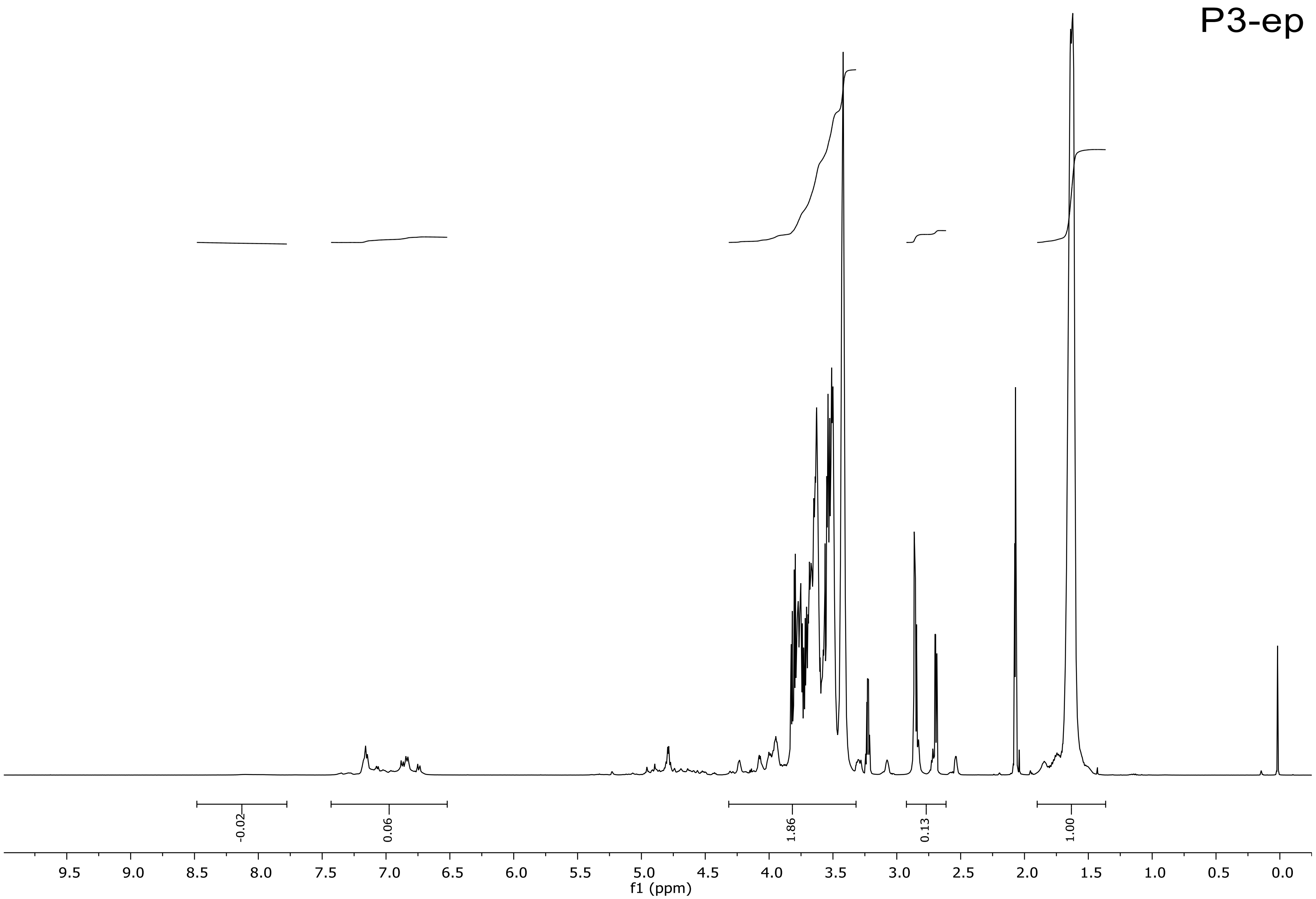

Figure S7. P3 epoxidized proton NMR.

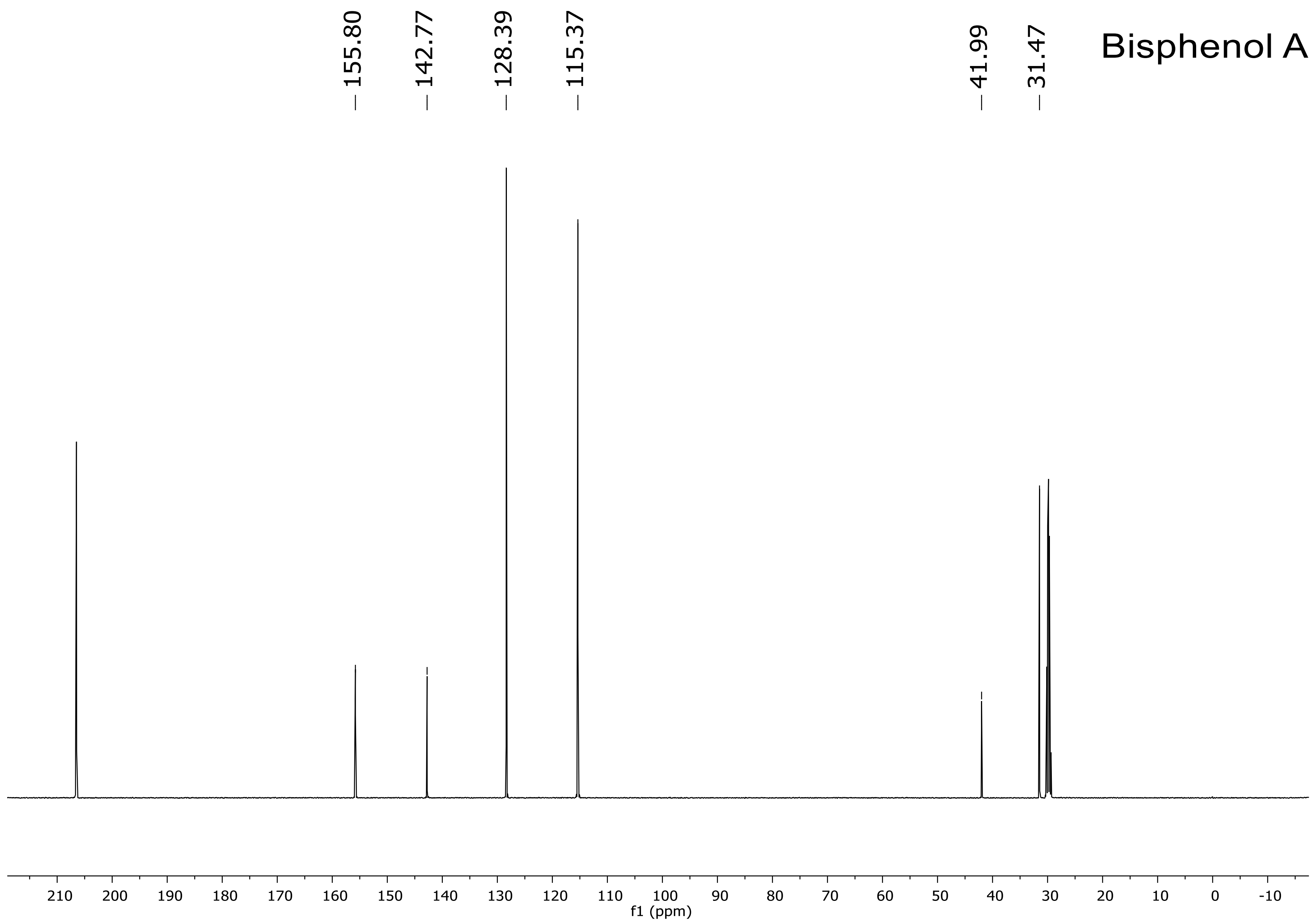

Figure S8. Bisphenol-A carbon NMR.

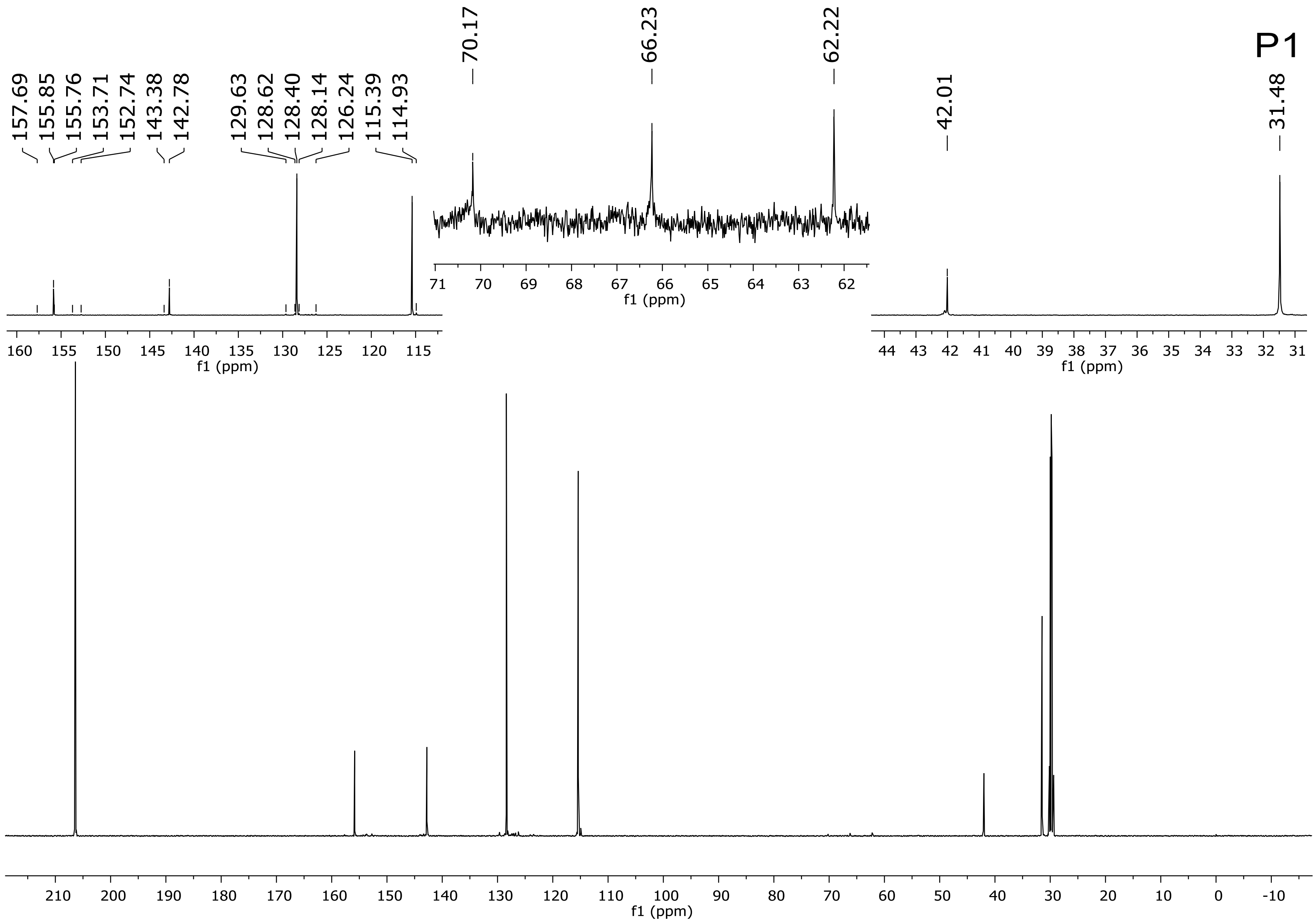

Figure S9. P1 carbon NMR.

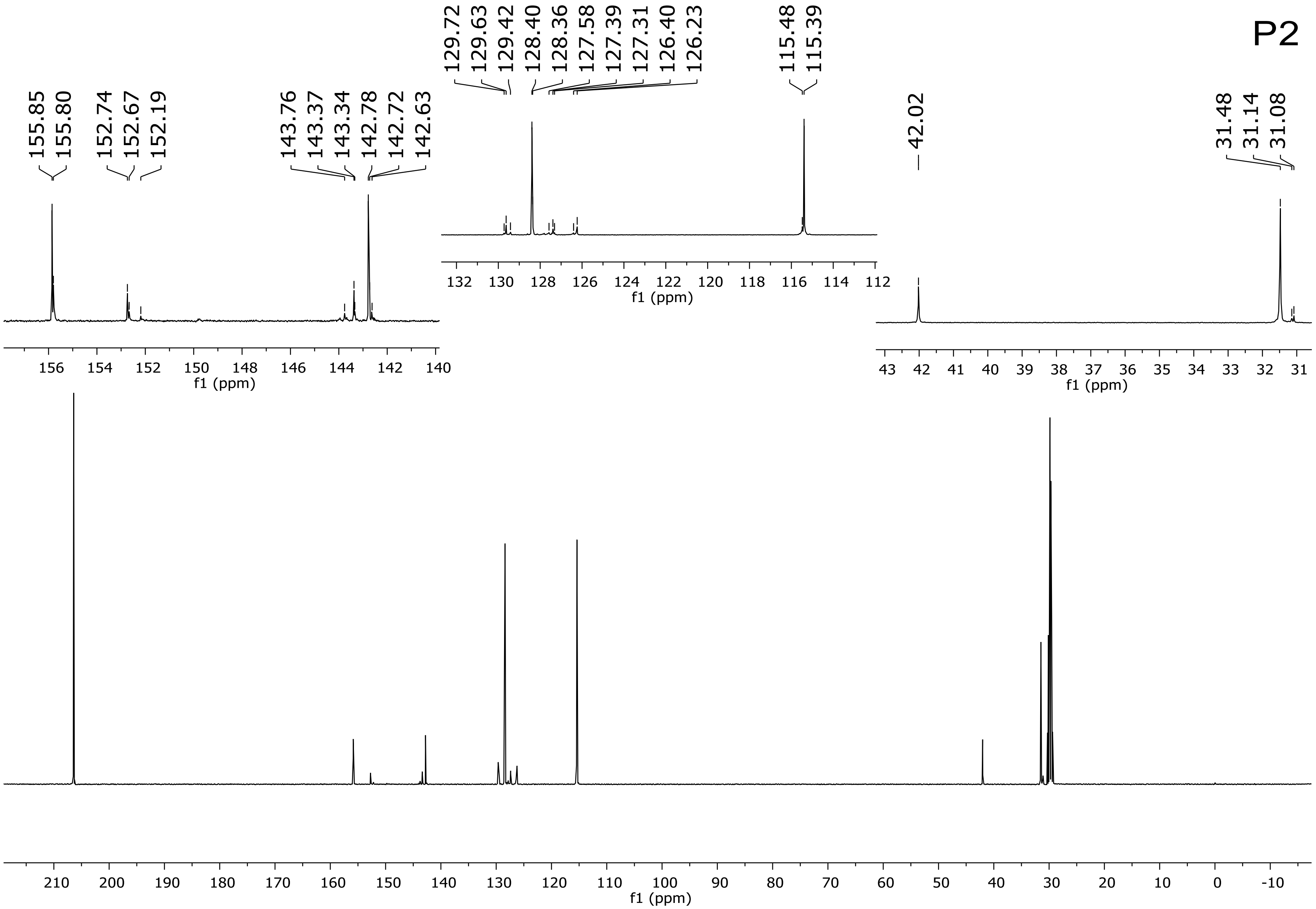

Figure S10. P2 carbon NMR.

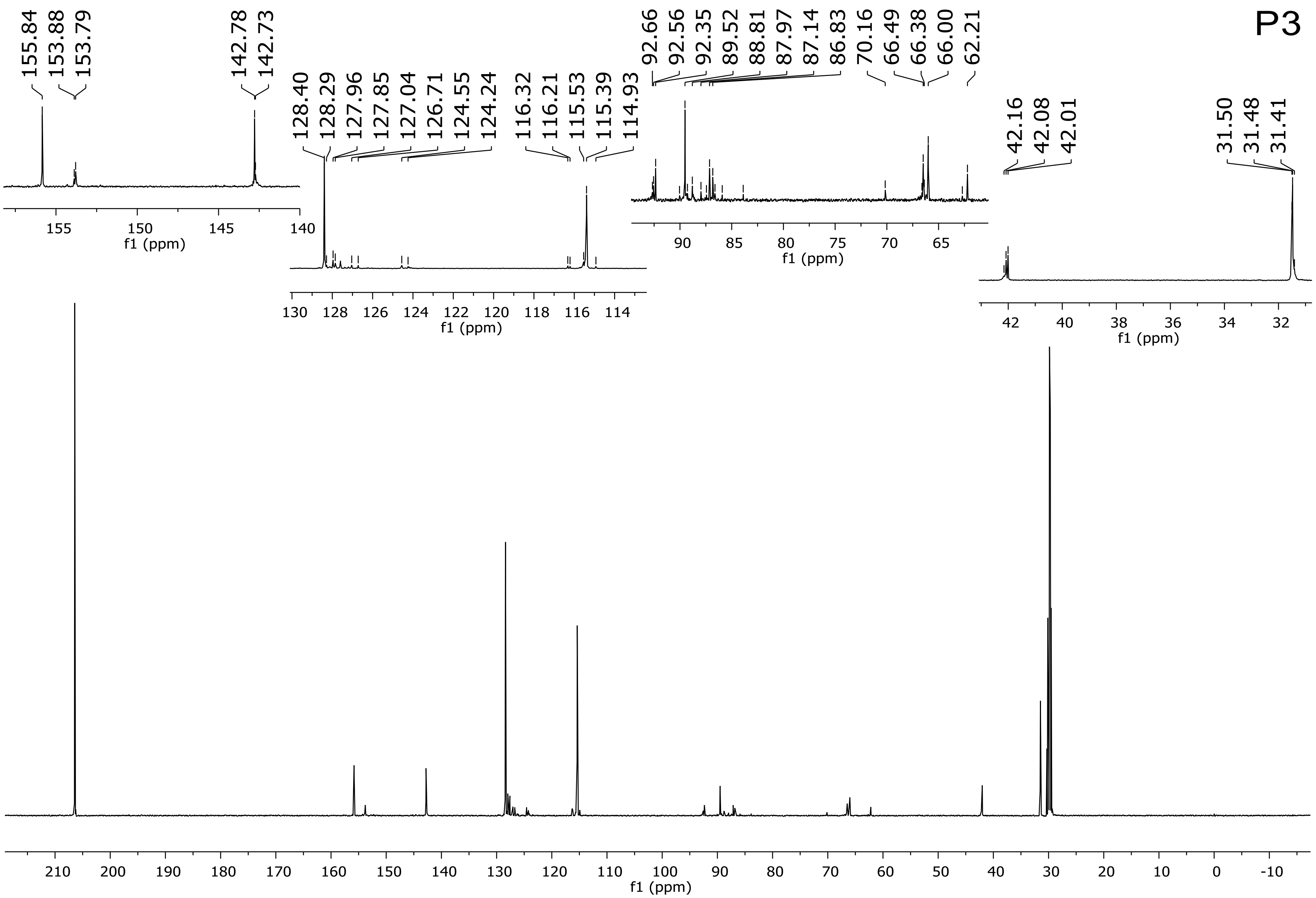

Figure S11. P3 carbon NMR.

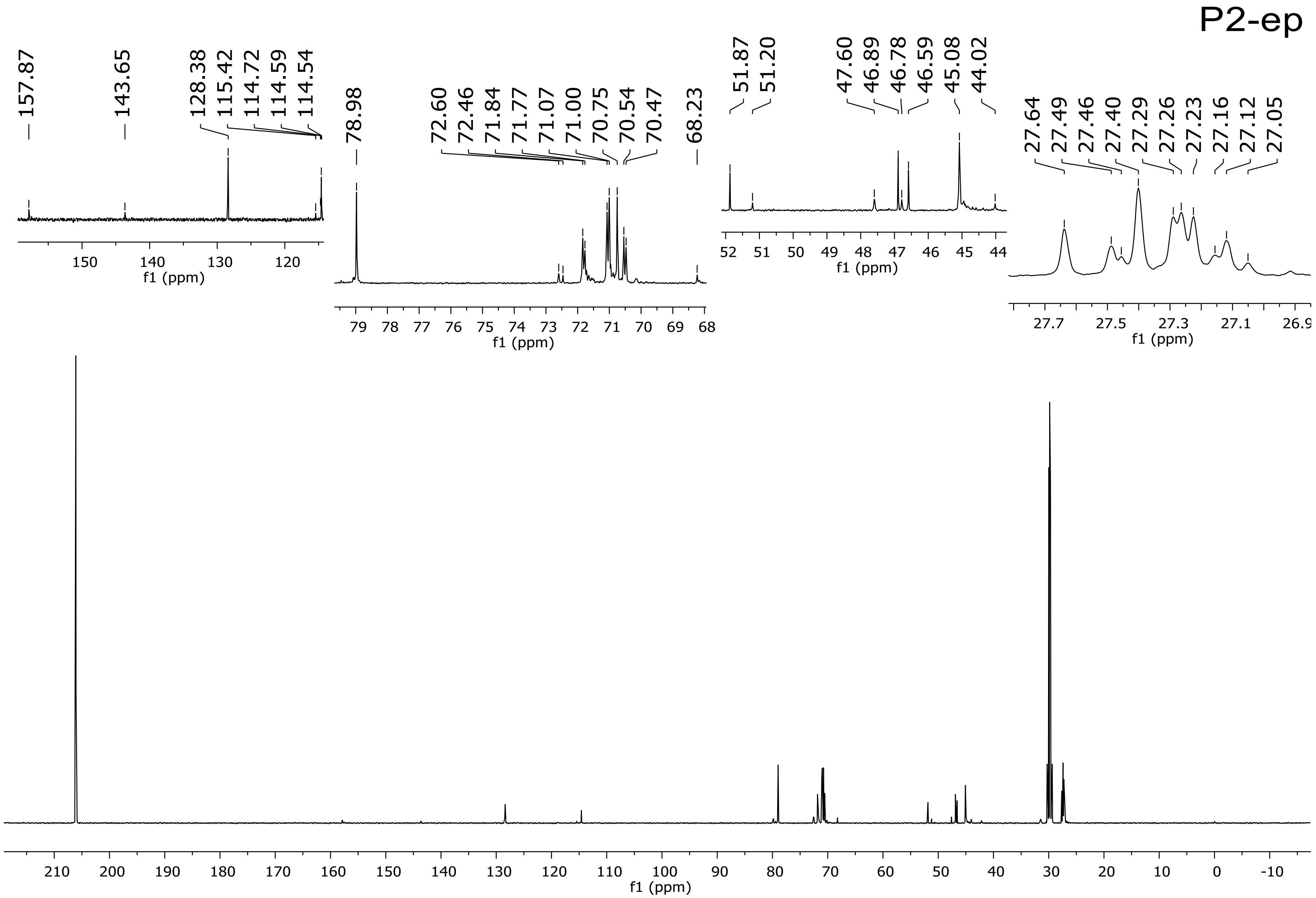

Figure S12. P1 epoxidized carbon NMR.

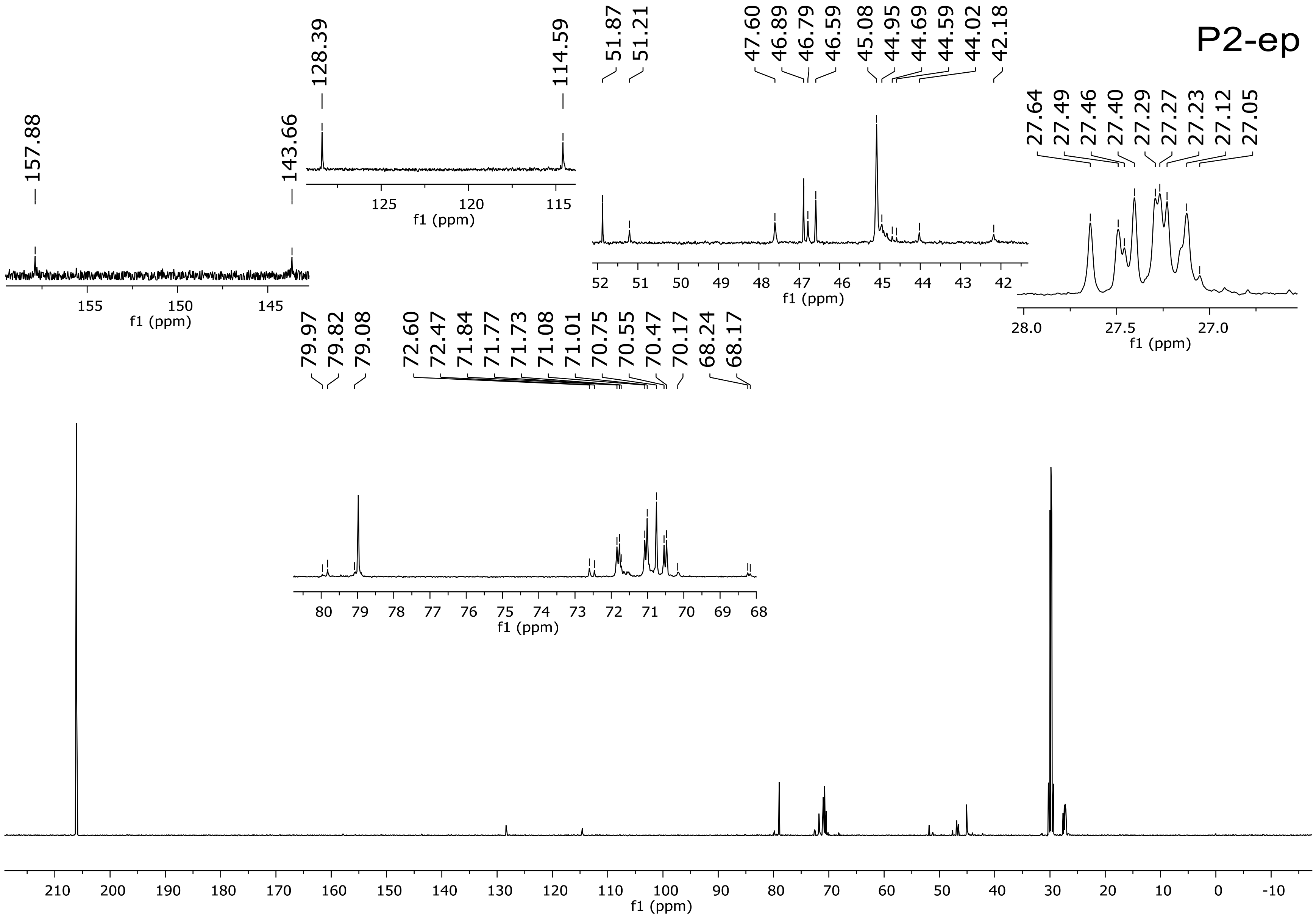

Figure S13. P2 epoxidized carbon NMR.

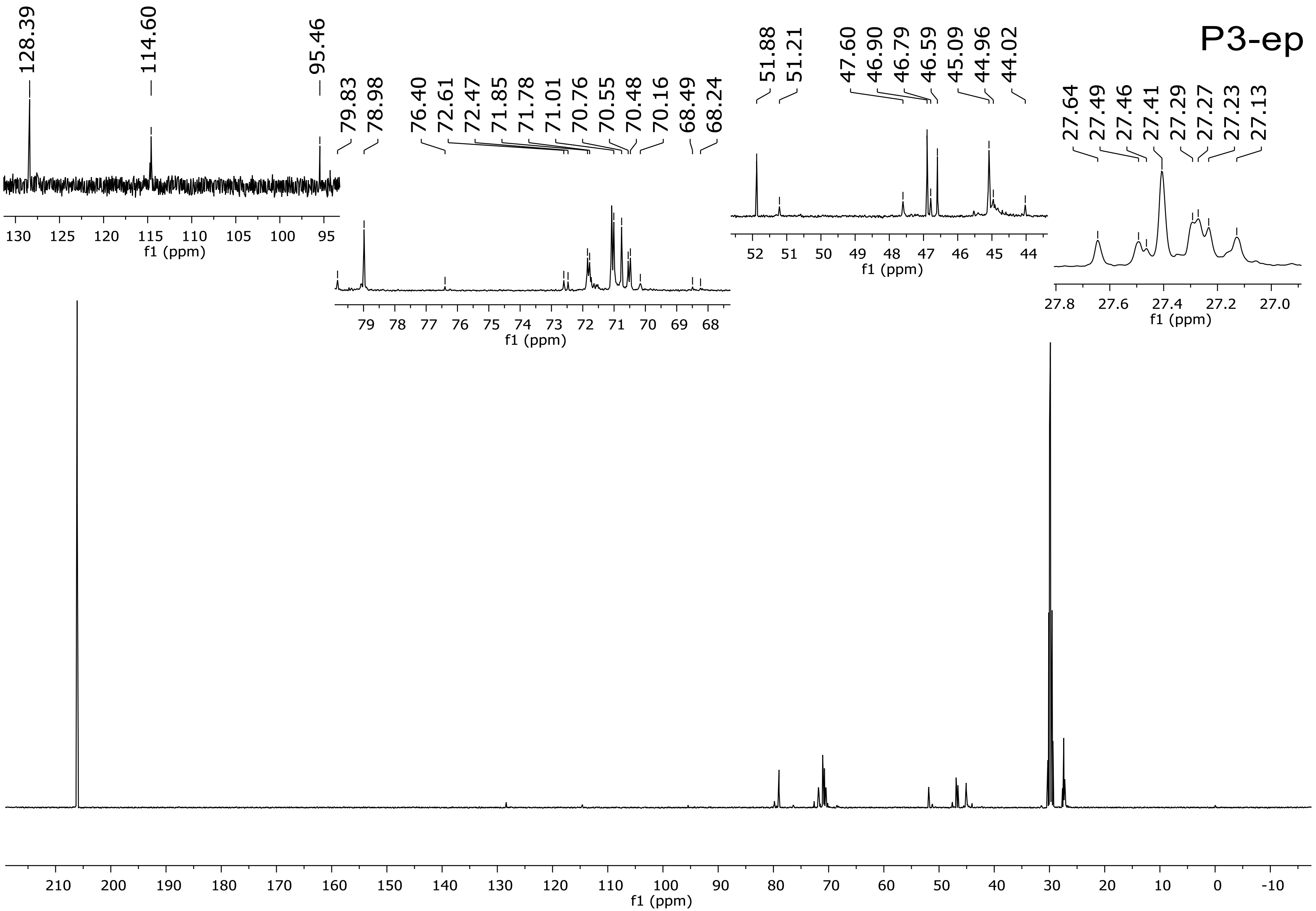

Figure S14. P3 epoxidized carbon NMR.

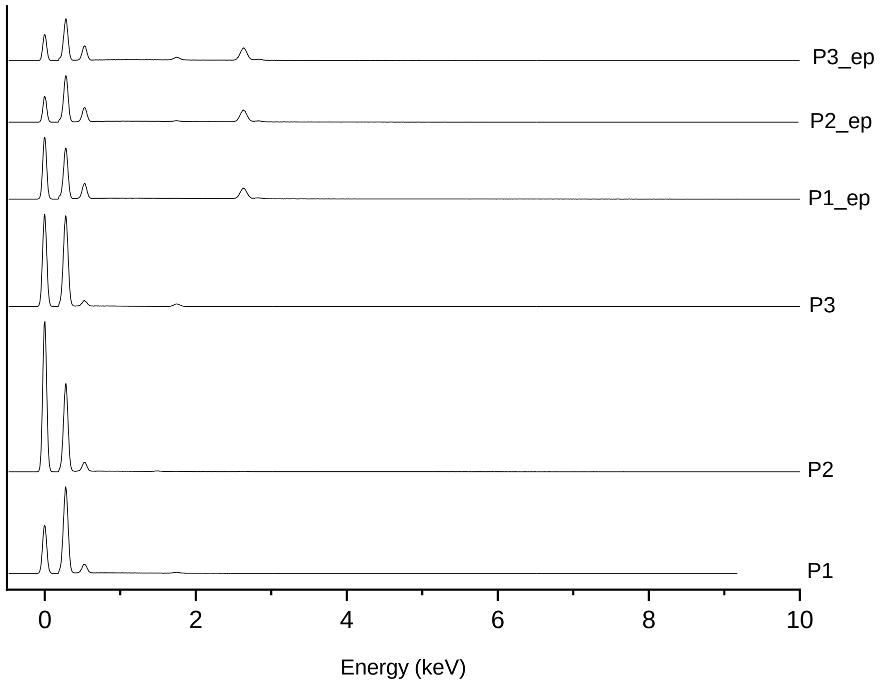

Figure S15. SEM-EDX elemental analysis for the samples P1 to P3 and P1_ep to P3_ep

Table S3: Relative atom percentage ratio for the non-epoxidized and epoxidized resins.

-	Relative % of atoms			
**Sample**	**Carbon**	**Oxygen**	**Chlorine**	**Silicon**
P1_a	84.26 (±8.0)	15.74 (± 2.6)	-	-
P1_b	84.36 (±8.0)	15.64 (± 2.6)	-	-
P1_c	84.21 (±8.0)	15.79(± 2.6)	-	-
P1-ep_a	75.80 (± 8.8)	18.21 (±2.7)	5.99 (±1.1)	-
P1-ep_b	75.53 (± 8.7)	19.82 (±3.0)	4.65 (±0.89)	-
P1-ep_c	76.03 (± 8.7)	19.24 (±2.9)	4.73 (±0.91)	-
P2_a	85.50 (±8.4)	14.09 (±2.4)	0.41 (±0.09)	-
P2_b	85.24 (±8.3)	14.39 (±2.4)	0.37 (±0.08)	-
P2_c	83.97 (±7.9)	16.03 (±2.7)	-	-
P2-ep_a	75.08 (± 8.7)	18.26 (±2.7)	6.39 (±1.2)	0.27 (±0.04)
P2-ep_b	74.94 (± 8.7)	19.25 (±2.9)	5.56 (±1.1)	0.24 (±0.04)
P2-ep_c	75.02 (± 8.6)	20.05 (±3.0)	4.94 (±0.94)	-
P3_a	85.76 (± 8.3)	13.89 (±2.3)	0.35 (±0.07)	-
P3_b	83.91 (± 8.2)	15.80 (±2.6)	0.29 (±0.06)	-
P3_c	84.25 (± 8.2)	15.38 (± 2.6)	0.37 (±0.08)	-
P3-ep_a	74.17 (±8.5)	20.24 (±3.0)	5.31 (±1.0)	0.28 (± 0.04)
P3-ep_b	72.98 (±8.4)	19.80 (±2.9)	5.84 (±1.1)	1.38 (± 0.21)
P3-ep_c	74.18 (±8.5)	20.31 (±3.0)	5.24 (±1.0)	0.26 (± 0.04)

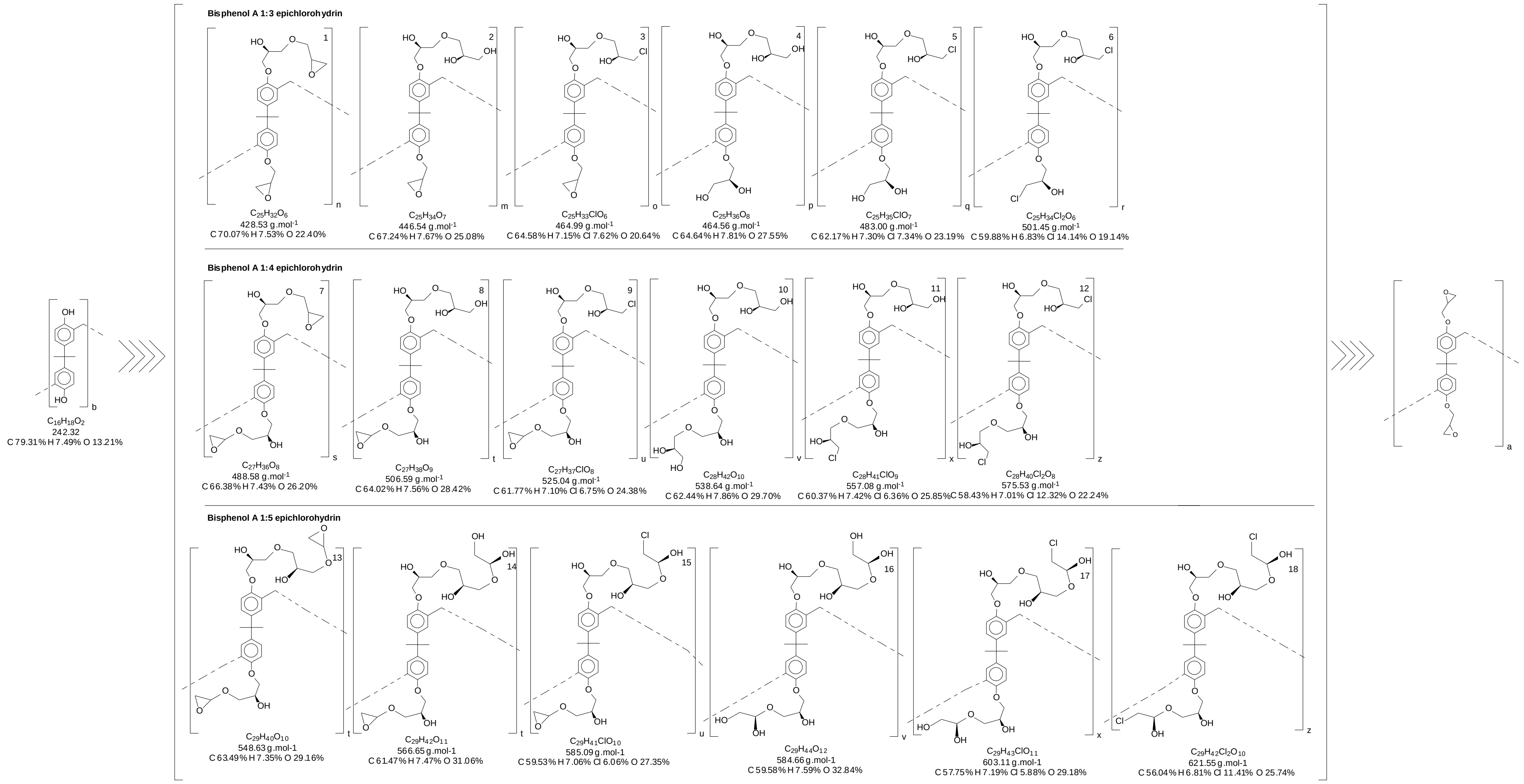

Figure S16. Possibilities of derivate during the alkylation reaction, considering the epoxidation degree until 5 units to one bisphenol-A.

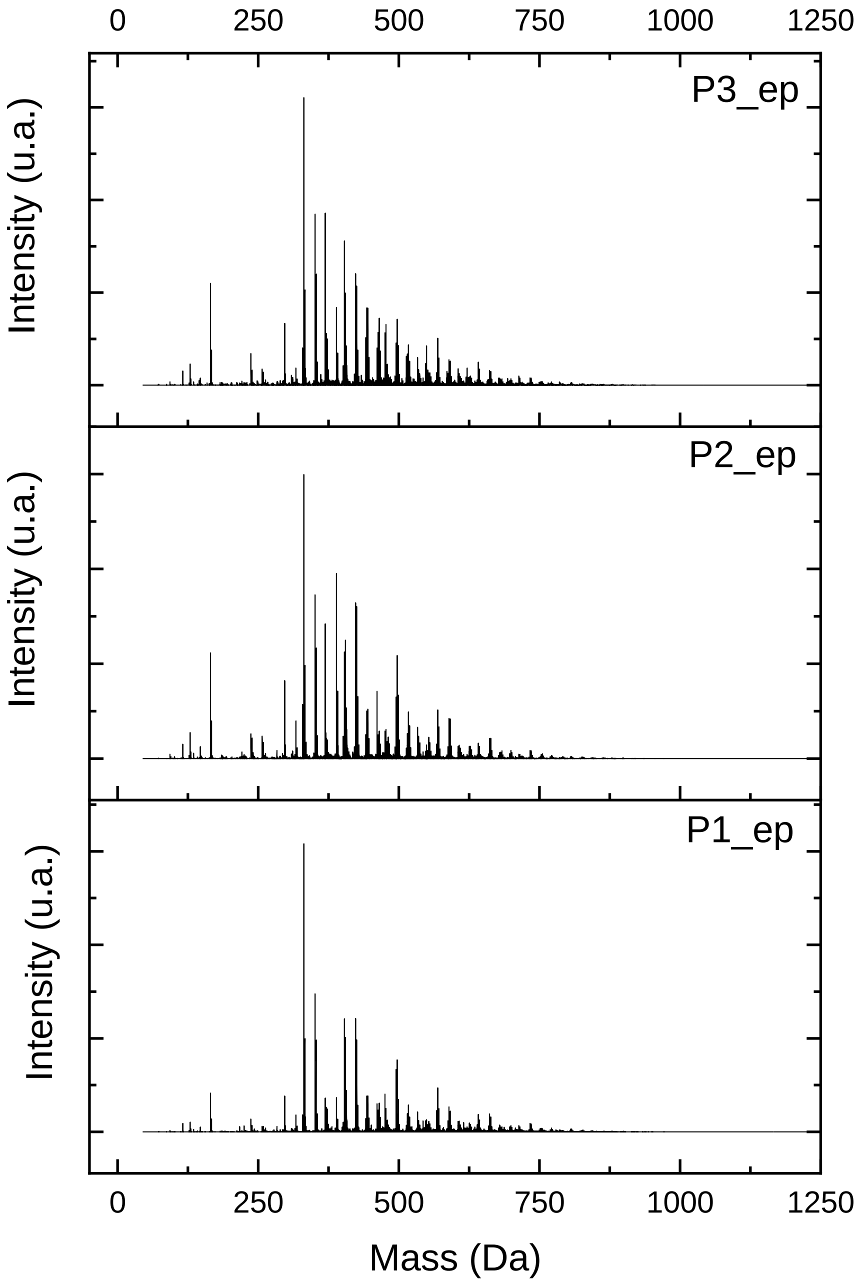

Figure S17. ESI-μ-TOF-MS spectra from the epoxidized resins.

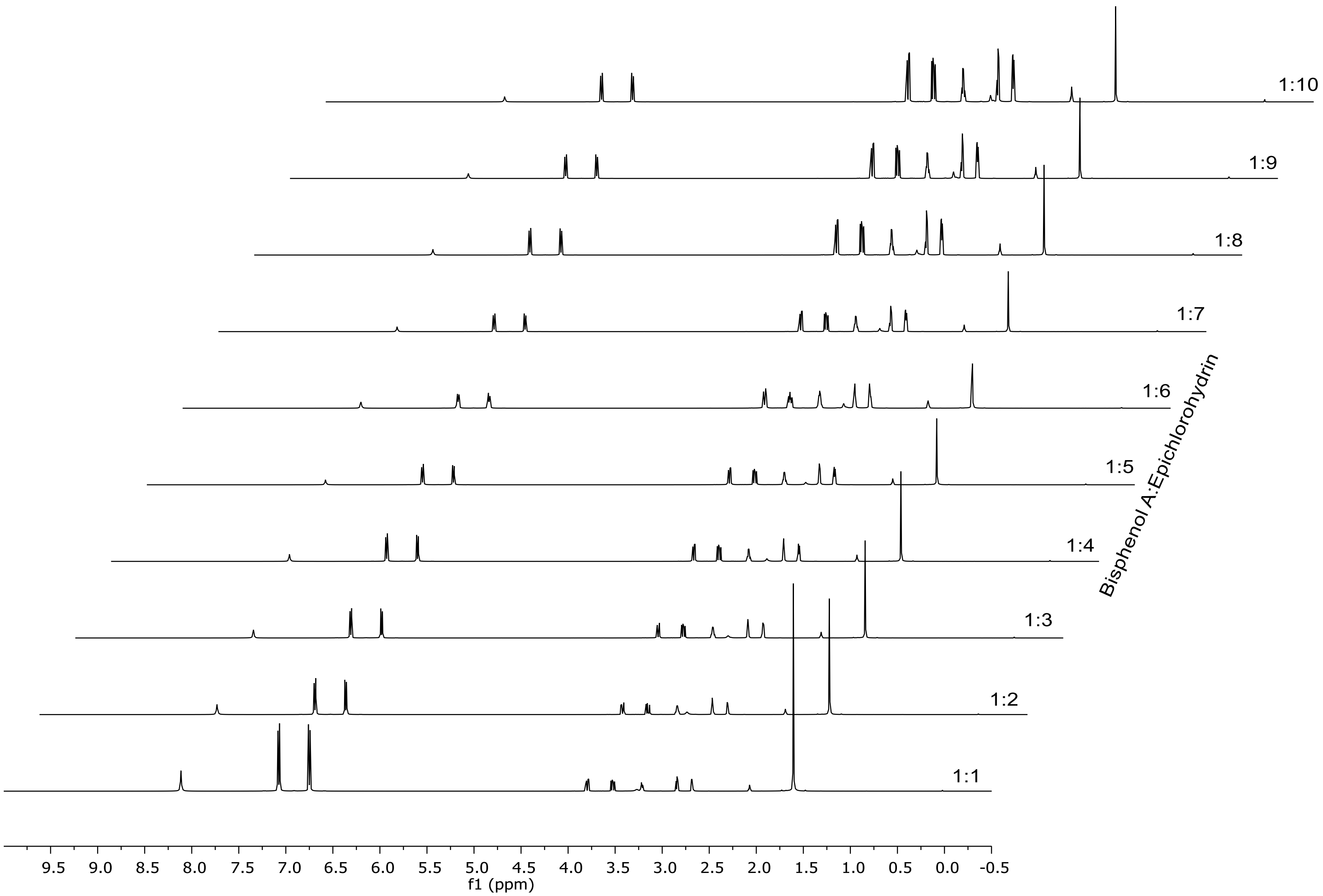

Figure S18. Bisphenol-A and Ephichlorohydrin from 1:1 to 1:10 molar ratio respectively.
